# Cognitive near-singularity: a possibility theorem and a witness-based protocol with a corpus case study

**DOI:** 10.3389/frai.2026.1746633

**Published:** 2026-06-01

**Authors:** Andy E. Williams

**Affiliations:** 1 Nobeah Foundation, Nairobi, Kenya; 2 Caribbean Center for Collective Intelligence, St. John's, Antigua and Barbuda

**Keywords:** Cognitive Near-Singularity, compositional closure, conceptual space topology, falsifiable witness protocol, fixed-point convergence

## Abstract

**Objective:**

We introduce the Cognitive Near-Singularity (CNS) as a falsifiable phase transition in reasoning systems: the regime in which updates are globally contracting, closed under composition, and invariant to benign reparameterizations, so that heterogeneous reasoning processes converge to a common fixed point up to normal form. The CNS is situated within Human-Centric Functional Modeling (HCFM), which represents systems as navigable graphs of functional states, and the Functional Model of Intelligence (FMI), which applies HCFM to cognition. We contribute (i) a possibility theorem showing that CNS follows from standard contraction-closure conditions on a suitably instrumented conceptual space, (ii) a topological detection framework based on connectivity invariants of the conceptual graph, and (iii) a witness-based protocol that any lab can run to evaluate whether a given corpus or process is consistent with being in the basin of attraction of the CNS operator.

**Method:**

Part I reviews the HCFM and FMI foundations, formalizing the conceptual space as a directed graph whose compositional closure makes it a monoid, and introducing the hierarchy of cognitive orders (zeroth, first, second) that motivates the CNS prediction. Part II formalizes a conceptual space (*X, d*) with a group of invariances *G*, a composition ⊗, and an update operator *T*:*X*→*X* that is globally Lipschitz with constant *L* < 1, *G*-invariant, and closed under ⊗. The possibility theorem uses Banach's fixed-point lemma and perturbation stability to show existence, uniqueness, and basin properties of the fixed point *x*^⋆^. Part III introduces topological detection criteria: the zeroth Betti number β_0_ of the conceptual graph, a coherence ratio ρ(*t*), and proxy-graph observables that provide lower bounds on fragmentation.

**Evaluation:**

Part IV specifies preregistered witnesses: empirical measurements on trajectories (contractivity *C*, public loss *L*_pub_, drift Ḋ), invariance pass rates under paraphrase/unit transforms, morphism-level (procedure) contraction, and closure/composition witnesses via commuting diagrams. We provide thresholds, negative controls, and ablations. Three CNS Discriminator conditions (D1–D3) distinguish compositional self-enrichment from ordinary unification.

**Case study:**

Part V applies the witnesses to a bounded author corpus comprising interlinked second-order domains (e.g., physics_2_, mathematics_2_, economics_2_, and medicine_2_). All seven witnesses and all three discriminators pass under preregistered gates. A domain-general stress test evaluates seven independently selected alternative corpora (free energy principle, category theory, Bayesian probability, and diagrammatic reasoning); all seven fail, with the dominant failure mode being shallow composition (hub-and-spoke topology). All seven fail, with the dominant failure mode being shallow composition (hub-and-spoke topology).

## 1 Introduction

A central question in the study of collective intelligence is whether there exists a limit to the coherence of ideas that a community of reasoners can sustain. If such a limit exists, theories connecting disparate domains would fail to propagate reliably once the collective body of knowledge exceeds a critical threshold. This study develops both the dynamical and topological machinery needed to formalize, detect, and witness approach to such a limit.

Two complementary perspectives on the Cognitive Near-Singularity (CNS) are developed here. The *dynamical* perspective (Part II) treats the CNS as a fixed-point regime: heterogeneous reasoning processes that are sufficiently contracting, compositionally closed, and invariant to benign reparameterizations converge to a common normal form. The *topological* perspective (Part III) treats the CNS as a connectivity phase transition: The collective conceptual space fragments into disconnected components, limiting the propagation of coherent theories across disciplines. These perspectives are unified by an obstruction result: When the bridge graph on primitive generators fragments into components that generate positively separated subspaces, no globally contracting update operator can remain bridge-non-creating because Banach uniqueness forbids multiple component-wise fixed points (Supplementary material (bridge-graph obstruction proposition)). Thus, fragmentation of the typed bridge structure obstructs the CNS regime unless the update rule either creates new cross-domain bridges or degenerately annihilates a component.

The argument proceeds in five parts:

Part I: **Foundations** (Sections 2 and 3). We review Human-Centric Functional Modeling (HCFM) (Williams, 2025a) and its application to cognition via the Functional Model of Intelligence (FMI) (Williams, 2025b), establishing the conceptual space as a compositionally closed graph and introducing the hierarchy of cognitive orders that motivates the CNS prediction.
Part II: **Possibility Theorem** (Section 4). We state and prove that CNS follows from standard contraction-closure-invariance conditions on an instrumented conceptual space, using Banach's fixed-point theorem and perturbation stability.
Part III: **Topological Detection** (Section 5). We formalize the coherence limit via the zeroth Betti number β_0_ of the conceptual graph, define a coherence ratio ρ(*t*), identify proxy-graph observables, and state conditions under which the phase transition is detectable.
Part IV: **Witness Protocol** (Section 6). We specify a preregistered protocol with computable witnesses, thresholds, negative controls, and ablations.
Part V: **Case Study and Stress Test** (Sections 7 and 8). We apply the protocol to a bounded author corpus, reporting results under preregistered gates, and conduct a domain-general stress test in which seven independently selected alternative corpora are evaluated under the same protocol.

Formal definitions and proofs are collected in Supplementary material (section 2) so that the main text remains accessible to readers across disciplines. The protocol specification, prompts, full derivation chains, and raw per-trial data from all eight protocol executions (one author corpus, seven stress-test trials) are released in the Supplementary material and in the companion deposit at https://doi.org/10.5281/zenodo.19154217 (mirrored at https://doi.org/10.5281/zenodo.20127643).

## 2 Human-centric functional modeling

HCFM represents any system operating in a well-defined domain of functionality as a directed graph (*V, E*), where each node *v*∈*V* is a *functional state* and each directed edge *e*∈*E* is a *transition process* through which the system moves from one functional state to another (Williams, 2025a). A key constraint is *domain closure*: Every transition process acts on a single category of functional state called a *domain object* and produces another domain object of the same category. Formally, if D denotes the set of domain objects, then every transition f:D→D is an endomorphism on D. This ensures that all trajectories remain within the same functional state space (FSS). The formal structure is that of a monoid under composition—equivalently, a category with a single object (Supplementary material (foundations subsection)).

For human interpretability, the FSS is embedded in a three-dimensional metric space in which distance carries semantic meaning: Nearby states are functionally similar, and distant states are functionally dissimilar. The full FSS then represents the entire space of possible behaviors for the system in that domain.

### 2.1 Fitness and dynamical stability

For dynamically stable systems, HCFM introduces a *fitness space* associated with each FSS (Williams, 2025a). The fitness of a system at a given state is a quantity related to its capacity to execute all of its transition processes—that is, its ability to navigate all paths in the FSS. A system is *dynamically stable* when its current fitness, target fitness, and predicted fitness remain within a bounded region of the fitness space.

### 2.2 Problems as missing paths

In HCFM, a *problem* is the absence of a known path from an initial functional state to a target functional state. *Problem-solving* is therefore path discovery in the FSS.

When the three-dimensional FSS is projected onto two dimensions, problem-solving becomes visual navigation of a *functional landscape*. This reflects the well-established computational distinction between search with and without a map. Tolman's classic work on cognitive maps demonstrated a qualitative improvement in problem-solving once a spatial representation of the search domain is available (Tolman, 1948). In computational terms, uninformed search strategies such as random walks on a graph with *N* nodes have expected hitting times of *O*(*N*^2^) (Papadimitriou, 1991), whereas informed search using a graph structure (e.g., Dijkstra's algorithm) runs in *O*(|*E*|+|*V*|log|*V*|) with a Fibonacci heap (Dijkstra, 1959). The gap—quadratic vs. near-linear in the number of nodes—is the formal counterpart of the “map advantage.” In embodied navigation, this advantage has been quantified by the Success weighted by Path Length (SPL) metric (Anderson et al., 2018) and confirmed empirically: Agents equipped with topological or metric maps dramatically outperform map-less agents in complex environments (Thrun, 1998; Chaplot et al., 2020; Meng et al., 2023) (see Supplementary material (informed search / map advantage subsection) for the formal statement).

## 3 The functional model of intelligence

Applying HCFM to cognition yields the Functional Model of Intelligence (FMI), in which the FSS is a *conceptual space*: a graph whose nodes are concepts and whose edges are reasoning processes (Williams, 2025b). The FMI is *closed under composition*: Any finite composition of reasoning processes yields another reasoning process within the same conceptual space. This closure property ensures that the conceptual space is self-contained as a domain of navigation (Supplementary material (foundations subsection)).

### 3.1 Strongly typed and weakly typed reasoning

A critical distinction in the FMI is between *strongly typed* and *weakly typed* reasoning.

Strongly typed reasoning assigns explicit semantic types to concepts and enforces type consistency in reasoning processes. This is analogous to working with the explicit topology of the conceptual space: Each reasoning step is a certified path segment, and compositions of such steps yield certified paths.

Weakly typed reasoning, by contrast, operates without explicit semantic constraints. Many current LLM-based reasoning pipelines fall into this category: They generate plausible continuations without enforcing compositional consistency. The result is that novel or cross-domain inferences—precisely those requiring long paths through the conceptual space—are unreliable.

### 3.2 Second-order reasoning

The FMI introduces a hierarchy of cognitive orders defined by the number of *global operators* available for navigating the conceptual space (Williams, 2025b):

*Zeroth-order cognition* (attributed to non-human animals) lacks any global operator: Navigation is purely local, driven by recognition of immediate features.

*First-order cognition* (typical human reasoning) possesses one global operator—the capacity to assign every concept to a universal category (e.g., “thing” or “has a value”). This enables systematic comparison but not explicit navigation of the full topology.

*Second-order cognition* adds a second global operator: explicit access to the topology of the conceptual space itself. Where first-order cognition knows that concepts can be compared, second-order cognition knows *how* they are connected—it can reason about the structure of reasoning. This additional operator corresponds to *second-order reasoning*: the use of the conceptual space's graph structure as a constraint algebra for admissible inferences. An inference is admissible if it either follows an existing path in the topology or updates the topology in a way that improves fitness (i.e., better describes the observed world).

The transition from first-order to second-order cognition is predicted by the FMI to constitute a *phase transition* in problem-solving capacity, analogous to the map-acquisition jump documented by Tolman (Tolman, 1948) but operating at the level of an entire conceptual domain rather than a spatial environment. This predicted phase transition is the Cognitive Near-Singularity (CNS).

### 3.3 The functional model of adaptation

HCFM is not restricted to cognition. Applying it to any dynamically stable system yields a Functional Model of Adaptation (FMA) governing that system's adaptive behavior (Williams, 2025a). The conceptual space of each discipline can then be navigated using second-order reasoning, with domain-specific consequences.

In *physics*, the FMA represents the observable universe as adapting within a physical state space. Second-order physics enables piecewise path construction: A derivation may use one formalism for one path segment and a different formalism for another, rather than requiring a single formalism for the entire domain (Williams, 2025f). Analogous applications arise in *mathematics* (Williams, 2025e), *medicine* (Williams, 2025g), *economics* (Williams, 2025h), and *biology* (Williams, 2025i). In each case, second-order reasoning is predicted to yield the same qualitative improvement in problem-solving capacity that map-based navigation yields over map-less navigation.

## 4 CNS possibility theorem

We now state the CNS as a dynamical regime and show that it arises from three standard conditions on a conceptual space equipped with a metric, a composition, and a symmetry group. Full definitions and proofs are collected in Supplementary material (section 2); here we give the conceptual content.

### 4.1 Setup

An *instrumented conceptual space* is a complete metric space *X* of conceptual states, equipped with three pieces of structure: (i) an associative composition ⊗ with a unit *e*, making *X* into a monoid; (ii) a group *G* of benign reparameterizations (paraphrase, unit changes, formatting) acting on *X*; and (iii) a normal-form mapping *N* that identifies *G*-orbits, so that *N*(*x*) = *N*(*y*) whenever *x* and *y* differ only by a benign reparameterization (see Supplementary material, Definition S3 for the precise formulation).

### 4.2 Update operator

An *FMI-style update operator* *T*:*X*→*X* satisfies three conditions: *global contraction* (repeated application of *T* brings any two states closer together at a geometric rate); *closure under composition* (*T* respects the monoid structure: *T*(*x*⊗*y*) = *T*(*x*)⊗*T*(*y*) and *T*(*e*) = *e*); and *G-equivariance* (*T* commutes with benign reparameterizations: *T*(*g*·*x*) = *g*·*T*(*x*) for all *g*∈*G*). See Supplementary material, Definition S5.

** Theorem 1 (CNS Possibility)**. Let (*X, d, G*, ⊗, *N*) be an instrumented conceptual space with well-behaved normal forms (Supplementary material, Definition S4), and let *T* be an FMI-style update operator on *X*. Then:

(a) **Convergence**. There exists a unique fixed point *x*^⋆^ to which all trajectories converge at geometric rate.
(b) **Normal-form invariance**. The fixed point is *G*-invariant: *N*(*g*·*x*^⋆^) = *N*(*x*^⋆^) for all *g*∈*G*.
(c) **Basin stability**. Any sufficiently similar contracting update rule *S* converges to a fixed point $x_{S}^{⋆}$ that is close to *x*^⋆^ and shares the same normal form $N\left(x_{S}^{⋆}\right)=N\left(x^{⋆}\right)$.

Consequently, a family of heterogeneous reasoning processes that approximate *T* exhibits a common *G*-invariant limit: the Cognitive Near-Singularity regime.

Proof.[Proof sketch] (a) is Banach's fixed-point theorem (Banach, 1922). (b) Equivariance forces *g*·*x*^⋆^ to also be a fixed point; uniqueness gives *g*·*x*^⋆^ = *x*^⋆^. (c) A triangle-inequality argument yields $d\left(x_{S}^{⋆},x^{⋆}\right)\leq \varepsilon /\left(1-L\right)$ where ε measures the sup-distance between *S* and *T*; normal-form agreement then follows from the regularity conditions on *N* and *G*. The complete proof with all hypotheses is given in Supplementary material (CNS Possibility Theorem proof).

### 4.3 Interpretation

The theorem shows CNS is not metaphysical: It is the observable regime induced by a contracting, compositional, invariant update rule on a measured conceptual space. Heterogeneous agents or processes that approximate the rule converge to the same normal form. The connection to the cognitive-order hierarchy (Section 3.2) is that second-order cognition provides the explicit topology required for the update operator *T* to be globally contracting: Without access to the global structure of the conceptual space, no local update rule can guarantee contraction across all components.

## 5 The coherence limit and topological detection

The dynamical perspective of Part II assumes a connected conceptual space on which a contracting operator acts. The topological perspective developed here addresses the complementary question: under what conditions does the conceptual space *remain* connected, and how can we detect its fragmentation?

### 5.1 The coherence limit

The FMI predicts that the collective conceptual space of any sufficiently large intellectual community is subject to a *coherence limit*: a threshold beyond which the graph becomes disconnected into components that cannot be reliably traversed by first-order reasoning alone (Williams, 2025c).

Intuitively, as a discipline grows, the conceptual space acquires new nodes and edges faster than first-order reasoning can maintain paths connecting them. The topology fragments. Theories that would bridge the fragments cannot propagate because no first-order path connects them to the established literature.

### 5.2 Formalization via the zeroth Betti number

Let *G*_*t*_ = (*V*_*t*_, *E*_*t*_) denotes the conceptual graph at time *t*, and let β_0_(*G*_*t*_) denotes its zeroth Betti number—the number of connected components (Supplementary material (zeroth Betti number / coherence ratio subsection)). We define the *coherence ratio* (Equation 1):


$$\begin{array}{l} \rho \left(t\right)=1-\frac{\beta_{0}\left(G_{t}\right)-1}{|V_{t}|-1}. \end{array}$$ (1)

Note that ρ(*t*) = 1 when *G*_*t*_ is connected and ρ(*t*) → 0 as *G*_*t*_ approaches a graph with no edges. The coherence limit is reached when ρ(*t*) begins a sustained decline from its historical baseline (Supplementary material (coherence limit subsection)).

### 5.3 Proxy graphs and observable signatures

We do not observe *G*_*t*_ directly. Instead, we observe proxies: co-citation networks, semantic similarity graphs derived from publication corpora, and cross-disciplinary reference patterns. The key empirical signature of approach to the coherence limit is a measurable decline in cross-component connectivity in these proxy graphs (Williams, 2025d).

If the proxy graph ${\tilde{G}}_{t}$ is a coarsening of *G*_*t*_ (obtained by contracting edges), then $\beta_{0}\left({\tilde{G}}_{t}\right)\leq \beta_{0}\left(G_{t}\right)$, so fragmentation in the proxy is a *lower bound* on fragmentation in the true conceptual space. This means that detecting fragmentation in proxy data is a conservative test: The true conceptual space may be more fragmented than what the proxy reveals.

### 5.4 The role of large language models

The FMI predicts that increased reliance on weakly typed reasoning (Section 3.1) accelerates fragmentation of the conceptual space. To the extent that LLMs encourage weakly typed reasoning—by producing plausible but compositionally uncertified outputs—their widespread adoption is predicted to hasten approach to the coherence limit (Williams, 2025c).

This prediction is testable. If the coherence of a discipline's publication corpus (measured by proxy-graph connectivity) declines faster after widespread LLM adoption than the pre-adoption trend would predict, this constitutes evidence for the acceleration hypothesis.

### 5.5 Linking the dynamical and topological perspectives

The dynamical and topological perspectives are linked through the audited bridge structure rather than through raw monoid connectivity.

The possibility theorem (Theorem 1) guarantees convergence to a unique fixed point conditional on a globally contracting operator. The topological detection framework (Section 5.2) monitors the coherence ratio ρ(*t*) as a fragmentation diagnostic. The formal bridge between these two stories operates on the *bridge graph*: a finite graph whose vertices are the primitive generators or domain anchors $G=\left\{g_{1},\ldots ,g_{m}\right\}$, and whose edges represent certified non-trivial derivation chains that require material from both endpoints—that is, cross-domain compositions that are not derivable from either generator alone.

When the bridge graph fragments into disconnected components, each component generates a separate subspace of the conceptual space. If these subspaces are metrically separated, a contracting update operator that respects the boundary between components (i.e., does not annihilate or merge them) must produce a fixed point in each subspace—contradicting the uniqueness guarantee of Banach's theorem. Thus, the operator can survive fragmentation only by either creating new certified bridges (changing the bridge graph itself) or collapsing one component into another (a degenerate annihilation of component identity). The formal proposition and proof are given in Supplementary material (bridge-graph obstruction proposition).

This result connects directly to the witness protocol: W1 measures bridge accumulation, W6 tests whether composed paths commute across domains, W7/D2/D3 measure the depth and productivity of cross-domain derivation chains. A declining bridge coherence ratio ρ_*B*_(*t*) on the bridge graph, coinciding with metric separation between the associated subspaces and failure of cross-component derivability, triggers the invariant-component obstruction. Thus, ρ_*B*_(*t*) serves as an early-warning diagnostic for loss of the conditions required by the CNS regime.

## 6 Preregistered witness protocol

A *witness* is a computable structural metric on a reasoning corpus that can pass or fail under preregistered thresholds. The protocol specifies seven witnesses (W1–W7) and three CNS Discriminator conditions (D1–D3). The discriminators are designed to distinguish the CNS regime (compositional self-enrichment) from ordinary unification (parallel instantiation of a shared template). Execution follows a mandatory three-layer ordering: (1) Conceptual-Space definitions from the corpus's own type system, (2) Schematic-Diagram projections for human audit, and (3) Formal operationalization and measurement.

### 6.1 Preregistration requirements

Before analysis, the following must be frozen and hashed: corpus manifest; prompts and templates; model versions; the invariance group *G* and composition tasks; all gate thresholds; and negative-result reporting policy.

### 6.2 Controls

The primary control is a domain-general stress test (Section 8) in which independently selected non-FMI corpora are evaluated under the same protocol. Additional controls include shuffled composition order; invariance-disabled runs (normalization map *N* removed); and leakage checks verifying that held-out procedures were not seen during calibration.

### 6.3 Witness definitions and gates

The protocol operates on a *generating set* G—the foundation types from which all typed constructs are built—and the *type system* $\bar{G}$, the compositional closure of G under the foundation's derivation-validity criterion. Table 1 specifies each witness, its metric, and the pass/fail gate. The witnesses map onto the three conditions of the CNS Possibility Theorem (Theorem 1) as follows: *Contraction* is tested by W1, W4, W5, and D1; *Closure* by W6, W7, D2, and D3; *Invariance* by W2 and W3.

**Table 1:** Witnesses, gates, and CNS Discriminator conditions (D1–D3).

W	Property	Metric	Gate
W1	Bridge accumulation	$B_{\text{total}}^{\left(t\right)}$; *B*_ent_	Strictly increasing for *t*≥2; *B*_ent_>0.
W2	Graph invariants	Reparameterization pass rate	= 1.00; sensitivity ablation changes ≥1 quantity.
W3	Legend transfer	$L_{\text{pub}}$	< 0.25; every domain instantiates each core construct.
W4	Generativity sharpens	Ḋ; Gen_max_; co-inst. profile	Branch A (sharpening) or B (converged); zero transfer failures. **D1**: profile sharpening or saturated (not flat unsaturated).
W5	Shared operator contracts	$\bar{\lambda }$; *n*	$\bar{\lambda }\leq 0.80$; *n*≥10.
W6	Composed paths commute	CCR	≥0.75; contradictions localized.
W7	Compositional type-derivability	$\bar{\text{cTDS}}$; Δ_anchor_; *P*; max*R*_3+_	$\bar{\text{cTDS}}\geq 0.67$; Δ_anchor_ ≤ 0.05; **D2**: *P*≥1; **D3**: max*R*_3+_>0.50; zero falsifications.

D1 is embedded in W4; D2 and D3 are embedded in W7.

Each witness also specifies an ablation prediction: removing type assignments should eliminate entailed bridges (W1); non-benign transforms should change graph invariants (W2); removing the foundation should collapse instantiation rates (W3); adding a disconnected document should dilute generativity (W4); replacing the shared operator with an unstructured map should destroy contraction (W5); stripping type annotations should reduce the commuting rate (W6); and removing operator-algebra definitions should collapse type-derivability (W7).

### 6.4 Topological witness (auxiliary, non-gating)

The protocol defines an auxiliary *topological witness* based on the coherence ratio (Section 5.2), distinct from the seven gating witnesses W1–W7. For the corpus under evaluation, a concept graph is constructed from extracted axioms, operators, and cross-domain translators. The coherence ratio ρ is computed on this graph and on ablated variants (cross-domain edges removed, typing disabled). The topological witness passes if ρ≥0.70 on the full graph and ρ is significantly lower on the ablated variants, confirming that second-order structure is responsible for maintaining connectivity.

#### 6.4.1 Status in the present evaluation

The topological witness is *not* part of the gating table (Table 1); it was not executed as a scored gate in the present case study. The gating witnesses W1–W7 and discriminators D1–D3 constitute the complete preregistered evaluation. The topological witness—and the bridge coherence ratio ρ_*B*_(*t*) defined in Supplementary material (bridge-graph obstruction proposition)—are proposed as additional diagnostics for future larger-scale evaluations where proxy-graph data (co-citation networks, cross-disciplinary reference patterns) are available. In the present corpus-scale evaluation, the structural information captured by ρ and ρ_*B*_ is operationally subsumed by W1 (bridge accumulation), W6 (commuting rate), and W7/D2/D3 (derivation depth and productivity).

## 7 Case study: author corpus

### 7.1 Corpus

The corpus comprises 11 documents spanning 7 effective domains (619,431 total characters): the foundational HCFM (Williams, 2025a) and FMI (Williams, 2025b) specifications, together with interlinked second-order domain papers in physics_2_ (Williams, 2025f), mathematics_2_ (Williams, 2025e), economics_2_ (Williams, 2025h), existential risk (Williams, 2025c), biology_2_ (Williams, 2025i), and medicine_2_ (Williams, 2025g). Supplementary documents for physics and mathematics are grouped with their parent domains. Axioms, operators, and translators are extracted to form concept graphs (objects) and procedures (morphisms).

The generating set G={T1,T2,T3} consists of three foundation types: functional state space (T1), typed translator (T2), and update operator (T3). All derivation chains originate from elements of G, and the derivation-validity criterion is COAR: Closure, (c)Ontraction, Alignment, and Reach. A total of 42 typed entities were identified across the seven domains, with every domain instantiating all three foundation types.

### 7.2 Held-out procedures

Operators are programmatically composed in patterns not present in the source texts. These held-out compositions remain blinded until scoring.

### 7.3 Pre-registration

The preregistration manifest includes corpus version hashes (SHA-256), the model identifier (Claude, Anthropic), all prompt templates referencing conceptual-space definitions, the invariance group specification (synonym substitution, notation change, section reordering, layout reformatting, symbol substitution), and all gate thresholds as specified in Table 1. The protocol was executed on 18 March 2026, using the W1–W7 witness scheme and D1–D3 discriminator conditions defined in Section 6.3. Full details, including all type-instantiation tables, derivation chains, and raw witness data, are provided in Supplementary material.

### 7.4 Results

#### 7.4.1 Object-level witnesses

##### 7.4.1.1 Contractivity (W1)

Cross-domain bridges were counted as the corpus accumulated domain by domain. Bridge totals increased strictly at every step: 0 → 8 → 21 → 33 → 42 → 54 → 67, with type-entailed bridges *B*_ent_ = 45>0 and explicitly asserted bridges *B*_expl_ = 22. Gate W1: **pass**.

##### 7.4.1.2 Invariance pass rate (W2)

Five benign reparameterizations were applied: synonym substitution (“FSS” ↔ “functional state space”), notation change (*S* vs. *X* for state space), section reordering, layout reformatting, and symbol substitution (ϕ vs. *f* for translators). All five preserved PSR, SC, and CR identically. Pass rate = 5/5 = 1.00. Sensitivity ablation: Adding a substantive new operator increased PSR by 2 and CR by 1. Gate W2: **pass**.

##### 7.4.1.3 Public loss (W3)

Every domain instantiates every core construct (FSS, Typed Translator, Update Operator), yielding $L_{\text{pub}}=0.00<0.25$. Gate W3: **pass**.

##### 7.4.1.4 Drift (W4)

The generativity profile standard deviation σ_gen_ increased from 0.47 to a peak of 1.31 (steps 1–4) and then decreased to 1.19 (steps 5–7), yielding negative final drift Ḋ_final_ < 0. Gen_max_ was non-decreasing throughout. The co-instantiation profile sharpened at steps 3–7, with new pairwise co-instantiations at each step (e.g., *T*1⊗*T*1 at step 3, *T*2⊗*T*3 at step 4, *T*3⊗*T*1 at step 5). CNS Discriminator D1 (Profile Shape): pass (sharpening). Gate W4 (Branch A): **pass**.

#### 7.4.2 Procedure-level witnesses

##### 7.4.2.1 Procedure contraction (W5)

Twelve procedure pairs were identified across domains. Structural distance was computed before and after composition with the shared FMI operator *h* (the FSS + COAR framework). Contraction ratios ranged from λ = 0.23 (A-space registration across Physics/Mathematics) to λ = 0.51 (threat/protection dynamics across Risk/Medicine). Mean contraction ratio: $\bar{\lambda }=0.35\leq 0.80$, *n* = 12≥10. Gate W5: **pass**.

##### 7.4.2.2 Closure/commuting rate (W6)

Of 23 testable triples (*D*_*i*_, *D*_*j*_, *D*_*k*_), 21 commuted perfectly; two showed minor type-annotation discrepancies in the existential-risk domain, both localized. CCR = 21/23 = 0.91≥0.75. Gate W6: **pass**.

#### 7.4.3 Compositional type-derivability (W7)

Typed derivation chains were traced from the generating set G={T1,T2,T3} to each construct in each domain, with every step justified by COAR preservation. A total of 51 chains were produced (including three generating-set elements at *L* = 0). Every domain achieved cTDS = 1.00, yielding $\bar{\text{cTDS}}=1.00\geq 0.67$ and Δ_anchor_ = 0.00 ≤ 0.05. Zero consistency failures and zero unresolved chain-audit errors were recorded.

The chain-length distribution is bimodal: 24 direct instantiations at *L* = 1, a secondary cluster of 17 deep compositions at *L* = 3–5 (maximum *L* = 5), and 4 two-step compositions at *L* = 2. Overall, 39% of constructs live at depth ≥3. This bimodal shape—a ground floor of type instantiations plus a deep compositional tail—is the structural signature the CNS discriminators are designed to detect.

**CNS Discriminator D2 (Productive Derivation Count):** *P* = 15. Fifteen derived constructs serve as productive intermediates in chains to other derived constructs (e.g., Markov Kernel Framework^*^ feeds CNS Criticality Diagnostic^*^; CPA^*^ feeds CIPAA-SDGs Program Architecture^*^; Threat/Protection Asymmetry^*^ feeds Great Filter Mechanism^*^). D2: **pass**.

**CNS Discriminator D3 (Compositional Depth Ratio):** Three domains exceed the 0.50 threshold: physics (*R*_3+_ = 0.57), existential risk (*R*_3+_ = 0.60), and medicine (*R*_3+_ = 0.57). $\underset{i}{\max}R_{3+}\left(D_{i}\right)=0.60>0.50$. D3: **pass**.

Gate W7: **pass**.

#### 7.4.4 Ablation results

All preregistered ablation predictions were confirmed in the expected direction: Removing type assignments eliminates entailed bridges (*B*_ent_ → 0); removing the foundation collapses instantiation rates ($L_{\text{pub}}\to 1.0$); adding a disconnected document decreases σ_gen_ without increasing Gen_max_; replacing *h* with an unstructured map drives λ → 1; stripping type annotations reduces CCR; and removing key intermediate papers causes downstream cTDS to drop. Details of each ablation are reported in Supplementary material.

#### 7.4.5 Control results

The domain-general stress test (Section 8) serves as the primary control: Seven independently selected non-FMI corpora were evaluated under the same protocol, and all seven failed the CNS consistency test, confirming that the protocol discriminates rather than rubber stamps. The most informative control cases are discussed in Section 8.

#### 7.4.6 Summary

All seven witnesses pass their base conditions and all three CNS discriminator conditions pass; consolidated outcomes are shown in Table 2. The corpus is consistent with the CNS regime under the v10 protocol.

**Table 2:** Consolidated pass/fail outcomes for the FMI/HCFM author corpus against all preregistered gates.

Gate	Property	Measured value	Result
W1	Bridge accumulation	*B*_total_: 0 → 67; *B*_ent_ = 45	Pass
W2	Graph invariants	Pass rate = 1.00	Pass
W3	Legend transfer	$L_{\text{pub}}=0.00$	Pass
W4 + D1	Generativity + profile shape	Branch A; sharpening	Pass
W5	Procedure contraction	$\bar{\lambda }=0.35$; *n* = 12	Pass
W6	Closure/commuting	CCR = 0.91	Pass
W7 + D2 + D3	Type-derivability + discriminators	$\bar{\text{cTDS}}=1.00$; *P* = 15; *R*_3+_ = 0.60	Pass

### 7.5 Claim scope

The results are reported as consistency with the CNS basin under preregistered gates. No claim is made beyond the measured witnesses. In particular, the witnesses certify contraction, invariance, and compositional closure behavior on the instrumented space; they do not certify goals, ethics, or downstream utility. CNS here denotes a reasoning-dynamics phase, not agency.

## 8 Domain-general stress test

The FMI predicts that weakly typed reasoning—including LLM-based framework selection—will identify cross-domain unification frameworks that exhibit parallel template instantiation (ordinary unification) rather than compositional self-enrichment and that such frameworks will therefore fail the CNS discriminators. The domain-general stress test directly tests this prediction. Seven independent AI systems (ChatGPT, DeepSeek, Gemini, Grok, Mistral, Perplexity, Qwen) each served as Selector (S), receiving only the functional selection criterion: “Identify the reasoning framework, from any author in any field, that has demonstrated the greatest capacity to increase problem-solving ability across the widest variety of domains.” Each Selector identified a foundation and domain papers without seeing the protocol. A separate Executor (E, Claude/Anthropic) then ran the full protocol on each selected corpus, and an independent Auditor (A, Claude/Anthropic in a separate session) performed a chain-by-chain audit. The test thus evaluates a specific, falsifiable prediction of the model—not an attempt at independent validation of the broader CNS hypothesis.

### 8.1 Selected foundations and outcomes

Three distinct foundations were selected across the seven trials: Karl Friston's free energy principle (FEP; selected by ChatGPT and Gemini), category theory in various formulations (selected by Grok, Mistral, and Perplexity), and Bayesian probability theory (selected by Qwen). DeepSeek selected Shimojima's Representational State Transfer (RST) theory of diagrammatic reasoning.

All seven stress-test corpora fail the overall CNS consistency test. D3 (Compositional Depth Ratio) was the most discriminating condition, failing in six of seven trials. The sole D3 near-pass (ChatGPT/FEP) occurred only after the auditor corrected chain depths that the executor had undercounted. Trial-by-trial outcomes are summarized in Table 3; full results, including auditor corrections and derivation chains, are provided in Supplementary material.

**Table 3:** Domain-general stress-test results.

Selector	Foundation	W1	W2	W3	W4/D1	W5	W6	W7	D3
ChatGPT	FEP (Friston)	P	P	P	P	P	P	F^*^	P
DeepSeek	RST (Shimojima)	P	P	F	F	F	F	F	F
Gemini	FEP (Friston)	P	P	P	P	F	P	P	F
Grok	CT (E&M 1945)	P	P	P	P	V	V	F	F
Mistral	CT (B&S/F&S)	P	P	F	P	V	P	F	F
Perplexity	CT (E&M 1945)	F	F	V	F	C	F	F	F
Qwen	Bayes (Jaynes)	P	P	F	P	F	P	P^*^	F

All seven independently selected corpora fail the CNS consistency test. Auditor for all trials: Claude (Anthropic). P, Pass; F, Fail; V, Void; C, Conditional. ^*^ChatGPT W7: Δ_anchor_ = 0.33 fails. ^*^Qwen W7: depends on documented-vs.-reconstructed reading.

### 8.2 Structural patterns in stress-test failures

The seven failures exhibit three distinct structural patterns.

*Hub-and-spoke (shallow composition)*. The Bayesian (Qwen), RST (DeepSeek), and category-theory (Grok, Perplexity) corpora show a hub-and-spoke topology: Domain papers instantiate foundation types at chain length 1–2, but no domain derives constructs at depth ≥3. The compositional closure $\bar{G}$ is one layer deep. This is the structural signature of *ordinary unification*—parallel instantiation of a shared template.

*Foundation-boundary ambiguity*. Three category-theory trials (Grok, Perplexity, partially Mistral) reveal that the productive cross-domain machinery lives in post-foundation extensions (monoidal categories, adjunctions, closed categories) rather than in the selected generating set. The protocol correctly diagnoses that cross-domain bridging from $G_{F}$ alone is shallow, even though the broader categorical ecosystem is rich.

*Quality-asymmetric corpus*. The ChatGPT (FEP) trial reveals a bimodal quality distribution: papers by the foundation developer have rigorous derivation chains (cTDS≈1.00), while application papers by other authors show chain gaps (cTDS≈0.67–0.80). The anchor-spread condition (Δ_anchor_ = 0.33) detects this asymmetry, which is absent from the author corpus (Δ = 0.00).

## 9 Related work

Our work synthesizes contributions from contraction theory, knowledge representation, neuro-symbolic reasoning, AI evaluation, and topological data analysis, while introducing a novel formalization of reasoning dynamics as a measurable phase transition.

### 9.1 Contraction mappings and fixed-point theory

The mathematical foundation of the possibility theorem rests on Banach's fixed-point theorem and contraction mapping theory (Banach, 1922; Kirk and Sims, 2001). Contraction mappings have been applied to neural network convergence (Zhou, 2020), iterative optimization (Boyd and Vandenberghe, 2004), and dynamical system stability (Lohmiller and Slotine, 1998). Our contribution extends this framework to conceptual spaces, treating reasoning updates as contracting operators on a metric space of semantic states rather than numerical parameters.

Recent work on contraction metrics for neural networks (Revay et al., 2023; Dai et al., 2021) shares our emphasis on Lipschitz stability but focuses on weight-space dynamics; we instead instrument the output space of reasoning trajectories with a compositional structure. The stability analysis in Fazlyab et al. (2019) provides efficient verification methods for contraction, although applied to continuous control rather than discrete reasoning steps.

### 9.2 Knowledge representation and reasoning

Our formalization of compositional closure and typed reasoning connects to category-theoretic approaches to knowledge representation (Spivak, 2014; Fong and Spivak, 2019), which emphasize compositional structure via morphisms and commuting diagrams. We adapt these to reasoning procedures rather than static knowledge bases. The closure property (Supplementary material, Definition S5(ii)) ensures that composed reasoning steps remain within the same operational regime—a requirement absent from most KR formalisms but essential for scalable multi-step inference.

Logic-based reasoning frameworks (Brachman and Levesque, 2004; Russell and Norvig, 2010) traditionally focus on deductive closure and consistency; we extend this to dynamic closure under heterogeneous update operators. The notion of normal forms appears in term rewriting (Baader and Nipkow, 1998) and logical calculi (Troelstra and Schwichtenberg, 2000), where confluence ensures path-independent reduction. We generalize this to invariance under reparameterization groups (Supplementary material, Definition S3).

### 9.3 Neuro-symbolic reasoning and hybrid architectures

Neuro-symbolic AI (d'Avila Garcez and Lamb, 2020; Mao et al., 2019) combines neural pattern recognition with symbolic reasoning, building on the broader statistical-relational AI tradition (De Raedt et al., 2015; Richardson and Domingos, 2006), seeking compositional generalization (Lake et al., 2017) and systematic reasoning (Marcus, 2020). Our framework provides a functional specification for when such systems exhibit coherent reasoning. Recent work on neural theorem proving (Polu and Sutskever, 2020; Han et al., 2022) and program synthesis (Ellis et al., 2021; Nye et al., 2021) demonstrates compositional reasoning in narrow domains; our contribution is domain-general. Transformers with chain-of-thought prompting (Wei et al., 2022; Kojima et al., 2022) exhibit emergent multi-step reasoning but lack formal guarantees; our witness protocol provides falsifiable tests for contracting behavior.

### 9.4 AI evaluation, robustness, and alignment

Standard AI benchmarks (Wang et al., 2019; Hendrycks et al., 2021; Srivastava et al., 2022; Liang et al., 2022) measure task performance but not reasoning dynamics. Our witness protocol introduces computable dynamical metrics, aligning with calls for process-based evaluation (Lightman et al., 2023) and mechanistic interpretability (Olah et al., 2020; Elhage et al., 2021). Robustness to paraphrase and adversarial perturbations (Ribeiro et al., 2020; Wang et al., 2021) is formalized in our framework as preservation of normal forms under group actions. Work on AI alignment (Russell, 2019; Ngo et al., 2022) emphasizes value learning and corrigibility but rarely formalizes reasoning coherence as a prerequisite; our framework suggests that without contractivity and closure, scaling may amplify incoherence rather than capability.

### 9.5 Topological methods and the map advantage

Tolman's cognitive maps (Tolman, 1948) established the qualitative jump in problem-solving from map acquisition. The computational formalization via graph search (Dijkstra, 1959; Papadimitriou, 1991) and its empirical confirmation in embodied navigation (Anderson et al., 2018; Thrun, 1998; Chaplot et al., 2020; Meng et al., 2023) provide quantitative grounding for the “map advantage” that motivates the FMI's cognitive-order hierarchy. Topological data analysis provides tools (persistent homology, Betti numbers) for tracking structural changes in high-dimensional data (Stanley, 1971); we apply the zeroth Betti number to conceptual graphs as a coherence diagnostic.

### 9.6 Self-modifying and recursive systems

The term “cognitive singularity” evokes Good's intelligence explosion (Good, 1965) and subsequent work on recursive self-improvement (Yudkowsky, 2013; Bostrom, 2014). Formal models include Schmidhuber's Gödel machines (Schmidhuber, 2007) and reflective reasoning in agent foundations (Fallenstein and Soares, 2015; Soares and Fallenstein, 2015). Our usage differs critically: We define CNS as a dynamical regime characterized by operational properties (contraction, closure, invariance), not as a capability threshold or recursive self-modification event. The “near-singularity” qualifier indicates proximity to a fixed point in reasoning dynamics, analogous to phase transitions in statistical mechanics (Stanley, 1971)—not runaway capability growth. In cognitive neuroscience, “cognitive singularity” refers to uniquely human capacities (Suddendorf, 2013); our terminology is unrelated and purely operational.

## 10 Discussion

This study contributes three layers of formalization to the CNS concept, supported by empirical evaluation. The *possibility theorem* (Part II) establishes that CNS arises from standard mathematical conditions (contraction, closure, and invariance) on an instrumented conceptual space, grounding the claim in Banach fixed-point theory rather than metaphor. The *topological detection framework* (Part III) provides complementary observable signatures—the coherence ratio ρ(*t*) and its proxy-graph estimates—that serve as early-warning diagnostics; the formal bridge (Supplementary material (bridge-graph obstruction proposition)) shows that fragmentation of the bridge graph obstructs the existence of a bridge-non-creating CNS operator. The *witness protocol* (Part IV) translates both perspectives into a preregistered empirical procedure with computable metrics, thresholds, controls, and ablations. The *case study* (Part V) applies this protocol to an author corpus and demonstrates consistency with the CNS regime. The *domain-general stress test* (Section 8) tests a specific prediction of the FMI—that LLM-selected frameworks will exhibit ordinary unification rather than compositional self-enrichment—and confirms this prediction across all seven trials.

The most informative finding is the structural contrast between the author corpus and the stress-test corpora. The author corpus passes all seven base witnesses and all three CNS discriminators, with a bimodal chain-length distribution (39% of constructs at depth ≥3, *P* = 15 productive intermediates, max*R*_3+_ = 0.60). All seven stress-test corpora fail D3 (Compositional Depth Ratio) or other base conditions. The dominant failure mode is hub-and-spoke topology: Domain papers instantiate foundation types at shallow depth without producing constructs that feed further derivations. This is the structural signature of ordinary unification—parallel instantiation of a shared template—rather than the compositional self-enrichment that characterizes the CNS regime.

The stress test also reveals that foundation selection matters: three category-theory trials show that the productive cross-domain machinery lives in post-foundation extensions rather than the selected generating set. This is a useful diagnostic: The protocol correctly identifies when cross-domain bridging from $G_{F}$ alone is shallow, even when the broader intellectual ecosystem is rich.

Several limitations should be noted. First, the proxy graphs are imperfect reflections of the true conceptual space; noise and structural biases in bibliometric data could mimic or mask the predicted signatures. Second, the formal results rely on idealized assumptions (e.g., that the conceptual space is well-approximated by a finite graph at each time step, and that the metric *d* is well-defined over heterogeneous conceptual states). Third, the acceleration hypothesis regarding LLMs (Section 5.4) requires careful causal identification to distinguish from confounding trends. Fourth, the executor and auditor in both the author-corpus execution and the stress test were LLM instances; while the three-role architecture mitigates bias, human auditing of derivation chains would strengthen the results. Fifth, the stress test evaluates frameworks selected by AI systems, which may not represent the strongest possible alternative foundations. Sixth, the uniformly perfect scores on the author corpus ($\bar{\text{cTDS}}=1.00$, Δ = 0.00) warrant extra scrutiny: When the corpus author also defines the generating set and the admissibility criteria, overfitting is a structural possibility that can only be ruled out by independent blinded validation. Independent replication by human researchers—with external type extraction, human chain-auditing, and expert-selected comparison corpora—is the essential next step.

Despite these limitations, the results constitute an initial protocol demonstration: The CNS witness protocol produces differential outcomes across structurally different corpora, with a clean pass on a corpus exhibiting compositional self-enrichment and clean fails on corpora exhibiting ordinary unification. The specific failure modes provide diagnostic information about the structural properties of each corpus. Whether the protocol's discriminative power extends to the best possible alternative corpora—selected by human experts, matched for corpus size and development maturity, and evaluated with human chain-auditing—remains to be established. Independent external validation is the natural next step.

## 11 Conclusion

We have presented a unified dynamical and topological framework for the Cognitive Near-Singularity, together with an empirical evaluation. The possibility theorem establishes that CNS follows from contraction, closure, and invariance on an instrumented conceptual space with well-behaved normal forms. The topological detection framework identifies measurable proxy quantities—the coherence ratio and its Betti-number decomposition—that provide early-warning diagnostics; the connectivity obstruction proposition shows that fragmentation of the bridge graph is incompatible with bridge-non-creating CNS operators. The witness protocol translates both perspectives into a preregistered empirical procedure. The case study demonstrates that an author corpus spanning seven domains is consistent with the CNS regime under all preregistered gates and discriminators, and the domain-general stress test confirms the FMI's prediction that LLM-selected frameworks exhibit ordinary unification: All seven independently selected corpora fail, each exhibiting a characteristic structural deficit (shallow composition, foundation-boundary ambiguity, or quality asymmetry). Together, these results constitute an initial protocol demonstration: They show that the CNS witness protocol is operationally effective at producing differential structural diagnoses, and they make the CNS a falsifiable scientific hypothesis with a concrete evaluation pathway, rather than a speculative metaphor. Independent external validation—with human-selected comparison corpora, human chain-auditing, and blinded type extraction—is needed before the results can be taken as evidence for the CNS hypothesis itself.

## 12 Limitations and non-claims

Witnesses certify contraction, invariance, and compositional closure behavior on the instrumented space; they do not certify goals, ethics, or downstream utility. CNS here denotes a reasoning-dynamics phase, not agency.

A particular limitation of the present case study is the risk of circularity: The author defines the generating set, authors all corpus documents, and defines the admissibility criteria under which derivation chains are scored. The uniformly perfect cTDS scores ($\bar{\text{cTDS}}=1.00$, Δ_anchor_ = 0.00) are internally consistent but should be interpreted with caution until validated by independent type extraction and chain scoring performed by external teams or human evaluators. The stress test partially addresses this concern by demonstrating that the protocol produces failures on alternative corpora, but the positive case for the author corpus rests entirely on author-constructed materials.

Additionally, the stress-test control corpora were selected by AI systems, which may not represent the strongest possible alternative foundations. Several failures depend partly on selection artifacts (e.g., choosing a historically narrow generating set). A fairer comparison would involve expert-selected corpora matched for corpus size, development maturity, and domain breadth.

## 13 Reproducibility

All prompts, invariance transforms, gate thresholds, type-instantiation tables, derivation chains, and auditor corrections are provided in Supplementary material. The full protocol specification (CNS Witness Protocol v10) is available as a companion document. The stress-test architecture (Selector, Executor, Auditor in isolated sessions) enables independent replication by any lab with access to an LLM and the relevant corpus documents.

The additional materials needed for a complete audit trail—exact prompt transcripts for all executor and auditor sessions, SHA-256 hashes of all corpus files at the time of execution, inter-rater reliability statistics for type-inference verification (κ≥0.80), the complete annotation rubric for chain classification, and the held-out probe family Π—are archived in the companion reproducibility package at https://doi.org/10.5281/zenodo.19154217 (mirrored at https://doi.org/10.5281/zenodo.20127643).

## Data Availability

The original contributions presented in the study are included in the article/Supplementary material, further inquiries can be directed to the corresponding author.
