# Supplementary material for: Cognitive near-singularity: a possibility theorem and a witness-based protocol with a corpus case study
Source: Front Artif Intell. 2026 Jun 1;9:1746633. doi: 10.3389/frai.2026.1746633 (PMC13267337; doi:10.3389/frai.2026.1746633)
Supplement: Supplementary file 1 [file Data_Sheet_1.pdf]

Supplementary Material

Cognitive Near-Singularity: Witness Protocol Execution
and Cross-Platform Stress-Test Results

Supplement to: "Cognitive Near-Singularity: A Possibility Theorem and a Witness-Based Protocol with a Corpus Case Study"

Anonymized for Peer-Review

March 2026

Abstract

This supplement provides the complete execution results for the Cognitive Near-Singularity (CNS) Witness Protocol v10. It contains two categories of results: (1) a full protocol execution on the author's FMI/HCFM corpus (11 papers, 7 domains), and (2) a domain-general stress test in which seven independent AI systems (ChatGPT, DeepSeek, Gemini, Grok, Mistral, Perplexity, Qwen) each selected the foundation they judged to have the greatest cross-domain problem-solving capacity, and a separate executor–auditor pipeline evaluated each selected corpus against the protocol. Corpus manifests, generating sets, type-instantiation tables, witness computations, derivation chains, gate outcomes, auditor corrections, and ablation results are reported here. The additional materials needed for a complete audit trail—exact prompt transcripts, SHA-256 hashes, inter-rater statistics, the annotation rubric, and the held-out probe family—are archived in the companion reproducibility package (see §28).

Contents

I Protocol Specification Summary 4

1 Architecture 4
- 1.1 Foundation Types . . . 4
- 1.2 Witness and Gate Definitions . . . 4
- 1.3 Witness-to-Theorem Mapping . . . 4
- 1.4 Stress-Test Architecture . . . 4
- 1.5 Reproducibility Requirements . . . 4
- 1.6 Scope and Non-Claims . . . 5

II Formal Apparatus 5

2 Functional State Spaces as Categories 6

3 Instrumented Conceptual Space: Full Definition 6

4 Connectivity and the Zeroth Betti Number 6

5 Detection Criterion 7

6 Complexity of Navigation 7

7 Proof of the CNS Possibility Theorem 7

8 Bridge Graph and Invariant-Component Obstruction 8

III Author-Corpus Execution 9

9 Corpus Manifest 9

10 Generating Set and Type Definitions 10

11 Type-Instantiation Extraction 10

12 Witness Results 12
- 12.1 W1: Contractivity (Bridge Counting) . . . 12
- 12.2 W2: Invariance (Graph Invariants) . . . 13
- 12.3 W3: Public Loss (Legend Transfer) . . . 13
- 12.4 W4: Generativity Sharpening and D1 (Profile Shape) . . . 13
- 12.5 W5: Procedure-Level Contraction . . . 13
- 12.6 W6: Closure / Commuting Rate . . . 14
- 12.7 W7: Compositional Type-Derivability and D2/D3 . . . 14
  - 12.7.1 Compositional Type-Derivability Scores . . . 14
  - 12.7.2 Chain-Length Distribution . . . 14
  - 12.7.3 CNS Discriminators . . . 14

13 Consolidated Gate Results (Author Corpus) 15

14 Ablation Predictions 15

IV Domain-General Stress Test 15

15 Overview of Stress-Test Trials 16

16 Trial 1: ChatGPT — Free Energy Principle 16

17 Trial 2: DeepSeek — Representational State Transfer 17

18 Trial 3: Gemini — Free Energy Principle 17

19 Trial 4: Grok — Category Theory 18

20 Trial 5: Mistral — Category Theory (Extended) 19

21 Trial 6: Perplexity — Category Theory (Historical) 20

22 Trial 7: Qwen — Bayesian Probability Theory 20

V Cross-Trial Analysis 20

23 Consolidated Comparison 21

24 Structural Diagnosis by Failure Mode 21

25 Discriminator Effectiveness **22**

26 Auditor Error Analysis **22**

27 Interpretive Caveats **23**

VI Replication Guide **23**

28 Materials Required **23**

29 Execution Procedure **24**

30 Interpreting Results **24**

31 Key Definitions for Replicators **25**

Part I
Protocol Specification Summary

This part provides a self-contained summary of the CNS Witness Protocol v10 sufficient for interpreting the results that follow. The full protocol specification is available as a companion document.

1 Architecture

The protocol measures seven structural witnesses (W1–W7) plus three CNS Discriminator conditions (D1–D3) to determine whether a reasoning corpus is consistent with the CNS regime: compositional self-enrichment, as opposed to mere parallel instantiation of a shared template.

Execution follows a mandatory three-layer ordering: (1) Conceptual-Space definitions from the corpus's own type system, (2) Schematic-Diagram projections for human audit, (3) Formal operationalization and measurement. All type vocabulary is foundation-native; no external vocabulary is imported.

1.1 Foundation Types

For any corpus, the protocol identifies a *generating set* $\mathcal{G}$—the finite set of foundation types from which all typed constructs are built. The *type system* $\overline{\mathcal{G}}$ is the compositional closure of $\mathcal{G}$ under typed, preservation-criterion-respecting derivation. A *typed derivation chain* is a finite sequence $T_1 \xrightarrow{\sigma_1} X_1 \xrightarrow{\sigma_2} \cdots \xrightarrow{\sigma_n} X_n$ where $T_1 \in \mathcal{G}$, each step preserves the foundation's derivation-validity criterion, and each step is auditable. Chain length is n.

1.2 Witness and Gate Definitions

1.3 Witness-to-Theorem Mapping

The seven witnesses map onto the three conditions of the CNS Possibility Theorem as follows: *Contraction* is tested by W1, W4, W5, and D1; *Closure* by W6, W7, D2, and D3; *Invariance* by W2 and W3.

1.4 Stress-Test Architecture

The domain-general stress test uses three independent LLM roles: Selector ($\mathcal{S}$), Executor ($\mathcal{E}$), and Auditor ($\mathcal{A}$). The Selector receives only a functional selection criterion (identify the framework with the greatest cross-domain problem-solving capacity) and does not see the protocol. The Executor receives the selected corpus and the protocol but not the Selector's rationale. The Auditor receives the Executor's output, the corpus, and the protocol, and performs an independent chain-by-chain audit.

1.5 Reproducibility Requirements

Each execution requires: corpus manifest with SHA-256 hashes; LLM model version and all prompts; type inference verification ($\geq 10\%$, $\kappa \geq 0.80$); reparameterization group G; held-out probe family Π; frozen gate thresholds; anchor domain identity; ablation predictions stated before execution; negative-result reporting policy; all raw data; prompts referencing conceptual-space definitions; co-instantiation profile at each step; P and R_{3+} per domain; and all stress-test transcripts.

Table 1: Consolidated gate table for the CNS Witness Protocol v10. D1, D2, D3 are the CNS Discriminator conditions that distinguish compositional self-enrichment from ordinary unification.

W	Property	Metric	Gate
W1	Bridges accumulate	$B^{(t)}_{\text{total}}$; B_{ent}	Strictly increasing for $t \geq 2$; $B_{\text{ent}} > 0$
W2	Graph invariants preserved	Reparameterization pass rate	$= 1.00$; sensitivity ablation changes ≥ 1 quantity
W3	Legend transfer	$\mathcal{L}_{\text{pub}}$	< 0.25; every domain instantiates each core construct
W4	Generativity sharpens	$\dot{D}$; $\text{Gen}_{\max}$; co-inst. profile	Branch A (sharpening) or B (converged); zero transfer failures; **D1**: profile sharpening or saturated (not flat unsaturated)
W5	Shared operator contracts	$\bar{\lambda}$; n	$\bar{\lambda} \leq 0.80$; $n \geq 10$
W6	Composed paths commute	CCR	≥ 0.75; contradictions localized
W7	Compositional type-derivability	$\overline{\text{cTDS}}$; Δ_{anchor}; P; $\max R_{3+}$	$\overline{\text{cTDS}} \geq 0.67$; $\Delta_{\text{anchor}} \leq 0.05$; **D2**: $P \geq 1$; **D3**: $\max R_{3+} > 0.50$; zero falsifications; zero unresolved chain-audit errors

1.6 Scope and Non-Claims

Results certify structural properties on the instrumented space. They do not certify the truth of domain-specific claims, intent, downstream utility, proximity to a CNS fixed point, or consciousness/agency. The CNS Discriminator conditions (D1, D2, D3) distinguish compositional self-enrichment from parallel instantiation. A corpus that passes W1–W7 base conditions but fails D1/D2/D3 demonstrates genuine unification without the self-enriching compositional closure that characterizes the CNS regime.

The results reported here constitute an *initial protocol demonstration*: they show that the protocol produces differential structural diagnoses across corpora with different compositional properties. They do not yet constitute independent empirical support for the CNS hypothesis, because the positive case rests on author-constructed materials evaluated by author-defined criteria, and the negative cases were selected by AI systems rather than human experts (see Section 27).

Part II

Formal Apparatus

This part collects the definitions and proofs supporting the main paper. The main-paper statements are *weakly typed*: they use accessible language and forward-reference this part for precise conditions. The definitions and proofs here are the *strongly typed* instances that validate those claims.

2 Functional State Spaces as Categories

Definition 1 (Functional State Space (Definition S1)). *A* functional state space *is a pair* $(\mathcal{D}, \mathcal{F})$ *where* $\mathcal{D}$ *is a set of domain objects (functional states) and* $\mathcal{F} \subseteq \{f : \mathcal{D} \to \mathcal{D}\}$ *is a set of transition processes satisfying:*

(i) ***Identity:*** $\mathrm{id}_{\mathcal{D}} \in \mathcal{F}$.

(ii) ***Closure under composition:*** *If* $f, g \in \mathcal{F}$, *then* $g \circ f \in \mathcal{F}$.

Remark 1. *Conditions (i) and (ii) make* $(\mathcal{D}, \mathcal{F})$ *a monoid under composition, and equivalently a category with a single object. The "completeness" of the FMI referenced in the main text is precisely this closure: no finite chain of reasoning steps escapes the conceptual space.*

Definition 2 (Conceptual Graph (Definition S2)). *The* conceptual graph *associated with* $(\mathcal{D}, \mathcal{F})$ *is the directed graph* $G = (V, E)$ *where* $V = \mathcal{D}$ *and* $(u, v) \in E$ *if and only if there exists* $f \in \mathcal{F}$ *with* $f(u) = v$.

3 Instrumented Conceptual Space: Full Definition

Definition 3 (Instrumented conceptual space (Definition S3)). *An* instrumented conceptual space *is a tuple* $(X, d, G, \otimes, N)$ *where:*

(i) (X, d) *is a complete metric space of conceptual states.*
(ii) G *is a group acting on* X *by isometries:* $d(g \cdot x, g \cdot y) = d(x, y)$ *for all* $g \in G$, $x, y \in X$.
(iii) $\otimes : X \times X \to X$ *is an associative composition with unit* $e \in X$.
(iv) $N : X \to \bar{X}$ *is a normal-form mapping identifying* G*-orbits:* $N(x) = N(g \cdot x)$ *for all* $g \in G$.

Definition 4 (Regularity conditions (Definition S4)). *An instrumented conceptual space has* well-behaved normal forms *if:*

(R1) ***Continuity:*** N *is continuous with respect to* d *and the topology on* $\bar{X}$.
(R2) ***Orbit separation:*** *Distinct* G*-orbits are metrically separated: if* $N(x) \neq N(y)$ *then* $\inf_{g \in G} d(g \cdot x, y) > 0$.
(R3) ***Local isolation:*** *Every* G*-orbit is locally isolated in the quotient: for each* $x \in X$, *there exists* $\eta(x) > 0$ *such that every orbit different from the orbit of* x *has all its elements at distance* $\geq \eta(x)$ *from* x.

Together, (R1)–(R3) ensure that sufficiently close points share the same normal form, and that the orbit of any fixed point has a definite separation radius from all other orbits.

Definition 5 (FMI-style update operator (Definition S5)). *An* update operator $T : X \to X$ *on an instrumented conceptual space satisfies:*

(i) ***Global contraction:*** $\exists\, L \in (0, 1)$ *such that* $d(Tx, Ty) \leq L\, d(x, y)$ *for all* $x, y \in X$.
(ii) ***Closure under composition:*** $T(x \otimes y) = T(x) \otimes T(y)$ *and* $T(e) = e$.
(iii) G***-equivariance:*** $T(g \cdot x) = g \cdot T(x)$ *for all* $g \in G$, $x \in X$.

4 Connectivity and the Zeroth Betti Number

Definition 6 (Zeroth Betti Number (Definition S6)). *For an undirected graph* $G = (V, E)$, *the zeroth Betti number* $\beta_0(G)$ *is the number of connected components of* G. *Equivalently,* $\beta_0(G) = \dim H_0(G; \mathbb{Z})$, *the rank of the zeroth simplicial homology group.*

For directed graphs, we work with the underlying undirected graph obtained by forgetting edge orientations (weak connectivity).

Definition 7 (Coherence Ratio (Definition S7)). *For a time-indexed family of graphs $\{G_t\}_{t\geq 0}$ with $G_t = (V_t, E_t)$, the coherence ratio at time t is*

$$\rho(t) = 1 - \frac{\beta_0(G_t) - 1}{|V_t| - 1}\,.$$

5 Detection Criterion

Proposition 1 (Fragmentation Signature). *Let $\{G_t\}$ be a family of conceptual graphs with $|V_t| \to \infty$. Suppose there exists a critical time t^* such that:*

(i) For $t < t^*$, the coherence ratio satisfies $\rho(t) \geq \rho_0$ for some constant $\rho_0 > 0$.

(ii) For $t > t^*$, $\rho(t) \to 0$ as $t \to \infty$.

Then the family $\{G_t\}$ undergoes a connectivity phase transition at t^*.

Remark 2. *In practice, we observe a proxy graph $\tilde{G}_t$ rather than G_t itself. If the proxy is a coarsening of G_t (obtained by contracting edges), then $\beta_0(\tilde{G}_t) \leq \beta_0(G_t)$, so fragmentation in the proxy is a lower bound on fragmentation in the true conceptual space.*

6 Complexity of Navigation

The "map advantage" cited in the main paper rests on the following standard results.

Proposition 2 (Search Complexity). *Let $G = (V, E)$ be a connected graph with $|V| = N$.*

(i) ***Uninformed search (random walk):*** *The expected time to reach a target node from a given source is $O(N^2)$ in the worst case.*

(ii) ***Informed search (Dijkstra):*** *Given the graph structure and non-negative edge weights, the shortest path can be found in $O(|E| + N \log N)$ time.*

The transition from (i) to (ii) is the formal analogue of acquiring a map of the search domain.

7 Proof of the CNS Possibility Theorem

We restate the theorem with fully explicit hypotheses and prove each part.

Full proof of Theorem 1 in the main paper. Hypotheses. $(X, d, G, \otimes, N)$ is an instrumented conceptual space (Definition S3) with well-behaved normal forms (Definition S4): G acts by isometries, N is continuous, distinct G-orbits are metrically separated, and each orbit is locally isolated. $T : X \to X$ is an update operator (Definition S5) with contraction constant $L \in (0, 1)$.

Part (a): Existence and uniqueness. Since (X, d) is a complete metric space and T is a contraction with Lipschitz constant $L < 1$, Banach's fixed-point theorem guarantees the existence of a unique $x^\star \in X$ satisfying $T(x^\star) = x^\star$. For any $x_0 \in X$, the iterates $x_{t+1} = T(x_t)$ satisfy

$$d(x_t, x^\star) \leq L^t\, d(x_0, x^\star),$$

which gives convergence at geometric rate L^t.

Part (b): Normal-form invariance. For any $g \in G$, the G-equivariance of T gives $T(g \cdot x^\star) = g \cdot T(x^\star) = g \cdot x^\star$. Thus $g \cdot x^\star$ is also a fixed point of T. By uniqueness (Part a), $g \cdot x^\star = x^\star$ for all $g \in G$, and hence $N(g \cdot x^\star) = N(x^\star)$.

Part (c): Basin stability. Define the sup-distance $\delta(S, T) = \sup_{x \in X} d(S(x), T(x))$.

Step 1: Fixed-point proximity. Let $S : X \to X$ be a contraction with constant $L_S < 1$ and $\delta(S,T) < \varepsilon$. By Banach's theorem, S has a unique fixed point $x^\star_S$. We bound the distance between the two fixed points:

$$d(x^\star_S, x^\star) = d(S(x^\star_S), T(x^\star)) \le d(S(x^\star_S), T(x^\star_S)) + d(T(x^\star_S), T(x^\star)) \le \delta(S,T) + L\, d(x^\star_S, x^\star).$$

Rearranging:

$$d(x^\star_S, x^\star) \le \frac{\delta(S,T)}{1-L} < \frac{\varepsilon}{1-L}.$$

Step 2: Normal-form agreement. By Part (b), the orbit of $x^\star$ under G is the singleton $\{x^\star\}$ (since $g \cdot x^\star = x^\star$ for all g). By condition (R3) of Definition S4, there exists $\eta = \eta(x^\star) > 0$ such that every element of every other G-orbit is at distance $\ge \eta$ from $x^\star$. Choose $\varepsilon < \eta(1-L)$, so that $\varepsilon/(1-L) < \eta$.

Since G acts by isometries (Definition S3(ii)), for each $g \in G$ the conjugated map $S_g = g \circ S \circ g^{-1}$ satisfies:

$$\delta(S_g, T) = \sup_x d(gS(g^{-1}x),\, Tx) = \sup_x d(gS(g^{-1}x),\, gT(g^{-1}x)) = \sup_y d(Sy, Ty) = \delta(S,T),$$

using isometry in the third equality and G-equivariance of T in the second. Since S_g is also a contraction (conjugation by an isometry preserves the Lipschitz constant), its unique fixed point is $g \cdot x^\star_S$. Applying Step 1 to S_g:

$$d(g \cdot x^\star_S, x^\star) \le \frac{\varepsilon}{1-L} < \eta \quad \text{for all } g \in G.$$

The entire G-orbit of $x^\star_S$ lies within the η-ball around $x^\star$. By definition of η (from (R3)), no orbit with a different normal form intersects this ball. Therefore $N(x^\star_S) = N(x^\star)$. □

8 Bridge Graph and Invariant-Component Obstruction

The following definitions and proposition make precise the claim in the main paper that fragmentation of the bridge structure obstructs the CNS regime.

A naive approach would define a "composition graph" on the full monoid $(X, \otimes, e)$ with an edge between x and y whenever $x = y \otimes z$ for some z. But in any unital monoid, $x = e \otimes x$ for all x, so every vertex is adjacent to the unit e and the graph is trivially connected. The correct object is the *bridge graph* on the finite set of generators, whose edges represent certified nontrivial cross-domain derivations.

Definition 8 (Bridge graph (Definition S8))**.** *Fix a finite generating family $\mathcal{G} = \{g_1, \dots, g_m\}$ of primitive foundation types or domain anchors. The* bridge graph $B = (\mathcal{G}, E_B)$ *has an edge (g_i, g_j), $i \ne j$, if and only if there exists an audited admissible derivation chain that uses material from both g_i and g_j and yields a construct not derivable from g_i alone or g_j alone.*

Definition 9 (Bridge-component subspace (Definition S9))**.** *For each connected component C_r of B, let $X_r \subseteq X$ be the closure (in the metric d) of all conceptual states obtainable by admissible typed derivations whose primitive support lies entirely in C_r.*

Definition 10 (Bridge-separated decomposition (Definition S10))**.** *The family $\{X_1, \dots, X_k\}$ (with $k \ge 2$) is a* bridge-separated decomposition *of X if:*
(A1) Each X_r is nonempty, closed, and complete in the induced metric.
(A2) The subspaces are pairwise positively separated: $\mathrm{dist}(X_r, X_s) \ge \sigma > 0$ *for all $r \ne s$.*
(A3) Admissible composition within X_r stays within X_r: if $x, y \in X_r$, then $x \otimes y \in X_r$.

Definition 11 (Bridge-noncreating operator (Definition S11)). *An update operator* $T : X \to X$ *is* bridge-noncreating *with respect to a bridge-separated decomposition if* $T(X_r) \subseteq X_r$ *for every* r*. Interpretation: the operator may refine states within a bridge-component, but it does not create certified cross-component bridges* ex nihilo.

Proposition 3 (Invariant-component obstruction). *Let* (X, d) *be a complete metric space with a bridge-separated decomposition* $\{X_1, \dots, X_k\}$ *satisfying* (A1)–(A3)*, with* $k \geq 2$*. Let* $T : X \to X$ *be a globally contracting map with Lipschitz constant* $L < 1$*. If* T *is bridge-noncreating, then* T *cannot exist.*

Proof. Each X_r is closed in the complete space X, hence complete. Since $T(X_r) \subseteq X_r$, the restriction $T_r = T|_{X_r} : X_r \to X_r$ is a contraction with the same Lipschitz constant $L < 1$. By Banach's fixed-point theorem, each T_r has a unique fixed point $x_r^\star \in X_r$.

Since $k \geq 2$, pick $r \neq s$. By (A2), $d(x_r^\star, x_s^\star) \geq \mathrm{dist}(X_r, X_s) \geq \sigma > 0$, so $x_r^\star \neq x_s^\star$. Thus T has at least two distinct fixed points in X, contradicting the uniqueness of the fixed point of a global contraction on a complete metric space. □

Corollary 1 (Fragmentation obstructs non-degenerate CNS). *If the bridge graph* B *is disconnected and its connected components induce a bridge-separated decomposition of* X*, then any globally contracting CNS operator must fail bridge-noncreation. Equivalently, a globally contracting update rule can survive bridge-graph fragmentation only by either:*

(i) creating new certified bridges *between components, thereby changing the bridge graph itself; or*

(ii) collapsing one component into another*, which annihilates the compositional identity of the collapsed component—a degenerate operation analogous to the constant-to-unit map* $T(x) = e$*, which maps every subspace into the unit's component.*

Definition 12 (Bridge coherence ratio (Definition S12)). *Let* B_t *be the audited bridge graph at time* t *on a fixed vertex set* $\mathcal{G}$*. The* bridge coherence ratio *is*

$$\rho_B(t) = 1 - \frac{\beta_0(B_t) - 1}{|\mathcal{G}| - 1},$$

where $\beta_0(B_t)$ *is the number of connected components of* B_t*.*

Remark 3 (Connection to the witness protocol). *The bridge coherence ratio* $\rho_B(t)$ *is directly observable from the quantities measured by the witness protocol: W1 (bridge accumulation) tracks* $|E_B|$*; W6 (commuting rate) tests whether bridges compose consistently; W7/D2/D3 measure the depth and productivity of the derivation chains that constitute the edges of* B*. A declining* $\rho_B(t)$*, coinciding with positive metric separation between the associated subspaces and failure of cross-component derivability, triggers the invariant-component obstruction (Proposition 3). Thus* $\rho_B(t)$ *serves as an early-warning diagnostic: it does not by itself prove contraction failure, but it identifies the structural condition under which the obstruction theorem applies.*

Part III
Author-Corpus Execution

This part reports the full execution of the CNS Witness Protocol v10 on the FMI/HCFM corpus. Executor: Claude (Anthropic). Date: March 18, 2026.

9 Corpus Manifest

The corpus comprises 11 documents spanning 7 effective domains (619,431 total characters):

Table 2: Corpus manifest. Supplementary papers are grouped with their parent domains.

#	Paper	Domain Label	Domain	Chars
1	HCFM v23	D_{F1}	Foundation	106,755
2	Intelligence from First Principles v31	D_{F2}	Foundation	83,442
3	FMA as Structural Unification for Physics v14	D_{Phys}	Physics	59,823
4	Supp. Info for FMA Physics v4	D_{Phys} (supp.)	Physics	16,358
5	Second-Order Mathematics (SOM) v20	D_{Math}	Mathematics	133,719
6	SOM CCL4 v5	D_{Math} (supp.)	Mathematics	76,883
7	SOM Supplementary Info v3	D_{Math} (supp.)	Mathematics	22,024
8	Application of 2nd-Order Methods: Econ Dev	D_{Econ}	Economics	32,073
9	Intelligence and the Great Filter v10	D_{Risk}	Existential Risk	57,281
10	Second-Order Biology	D_{Bio}	Biology	14,613
11	Second-Order Medicine and Healthcare	D_{Med}	Medicine	16,537

10 Generating Set and Type Definitions

The generating set $\mathcal{G} = \{T1, T2, T3\}$ consists of three foundation types. All derivation chains originate from elements of $\mathcal{G}$. The derivation-validity criterion is COAR: Closure, (c)Ontraction, Alignment, and Reach.

Definition 13 (T1: Functional State Space (FSS)). *A complete metric graph (S, d) carrying: (a) Contraction: $d(Tx, Ty) \leq L\, d(x, y)$, $L < 1$; (b) Closure: $T(x \otimes y) = T(x) \otimes T(y)$; (c) Invariance: $T(g \cdot x) = g \cdot T(x)$ for all $g \in G$; (d) Fitness geometry: $\phi : S \to \mathbb{R}$; (e) Complexity: $\ell : S \to \mathbb{R}_{\geq 0}$; (f) Alignment: coherence across G-orbits; (g) Normal form: canonical representative $N(x)$ per G-orbit; (h) Compositionality: states and transitions compose via $\otimes$ with unit e.*

Definition 14 (T2: Typed Translator). *$\phi : S_A \to S_B$ between two FSSs preserving: (a) type admissibility of compositions; (b) contraction; (c) closure.*

Definition 15 (T3: Update Operator). *$T : S \to S$ on an FSS carrying: (a) global contraction; (b) closure under composition; (c) G-equivariance; (d) fixed-point existence and uniqueness (Banach).*

11 Type-Instantiation Extraction

For each domain, entities were identified that carry explicit type assignments to one of the three foundation types. Table 3 provides the full extraction. A total of 42 typed entities were identified across 7 domains, with every domain instantiating all three foundation types.

Table 3: Type-instantiation extraction by domain. Each row records a domain entity, its assigned FMI type, and textual evidence.

Domain	Entity	Type	Evidence
D_F	Conceptual Space	T1	HCFM: "functional state spaces that represent the entire space of possible behaviors"
D_F	Cognitive Fitness Space	T1	HCFM: cognitive fitness space with scalar/vector fitness, attractors

Continued on next page

Domain	Entity	Type	Evidence
D_F	Awareness Space	T1	HCFM: Ω = Awareness; domain object, closure, contraction
D_F	External Functional Primitives	T3	HCFM/IFP: storage, recall, System 1, System 2; closed, contractive
D_F	Internal Functional Primitives	T3	IFP: 6 recursive functions; closure-preserving, G-equivariant
D_F	Markov Kernel Updates	T3	IFP: "formalize updates as Markov kernels"; Wasserstein contraction
D_F	MFM↔MAM Bridge	T2	HCFM: behavior-preserving, proof-carrying bridge
D_F	Cross-Domain Mapping (I6)	T2	IFP: internal primitive enabling cross-domain reasoning
D_F	Topology-Aware Folding	T3	IFP: monotone Betti reduction; preserves closure
D_{Phys}	P-space	T1	FMA: "strongly typed domain: P-space" with operator algebra
D_{Phys}	A(R)-space	T1	FMA: awareness/registration operators
D_{Phys}	FMA Specification v0.2	T2	FMA: typed specification as corpus-level drift-checker
D_{Phys}	UCCD Protocol	T2	FMA: Unified Coherence Constraint Diagnostic
D_{Phys}	Coarse-Graining Operators (Σ)	T3	FMA: Σ-mediated coupling; thermodynamic coarse-graining
D_{Phys}	FMI/CNS Operator	T3	FMA: Def 11.2 satisfying contraction, closure, invariance
D_{Math}	M-space	T1	SOM: tower of semantic manifolds; compositionally complete
D_{Math}	A-space	T1	SOM: synchronization/registration configurations
D_{Math}	Domain-FSS (Practice Space)	T1	SOM Supp: mathematical practice as trajectories
D_{Math}	Inter-Context Translators	T2	SOM: 1-morphisms with coherence conditions as 2-morphisms
D_{Math}	Compression/Expansion Operators	T3	SOM: primitive update operators on M-space
D_{Math}	CCA Gates	T3	SOM: governance analogues of Closure/Contraction/Alignment
D_{Econ}	Domain FSS	T1	EconDev: "each domain as a functional state space"
D_{Econ}	Translation Layer	T2	EconDev: "preserves invariants across domains"
D_{Econ}	CPA	T2	EconDev: combinatorial search engine across domains
D_{Econ}	Contraction Audits	T3	EconDev: Lipschitz constant of update operator measured

Continued on next page

Domain	Entity	Type	Evidence
D_{Econ}	Synchronization Operators	T3	EconDev: maximize coordinated action per unit cost
D_{Risk}	Civilizational Conceptual Space	T1	GF: "intelligence as navigation through a conceptual space"
D_{Risk}	Higher-Dimensional AI Spaces	T1	GF: accessible only to AI systems
D_{Risk}	Generalization Operator	T3	GF: "spans the entire conceptual space"
D_{Risk}	Cross-Domain Generalization	T2	GF: order transitions via abstraction, metaphor
D_{Risk}	Inward-First Containment	T3	GF: $\Pi_N = \chi_{\text{in}}^N$, $\chi_{\text{in}} < 1$
D_{Bio}	Cellular Regulatory FSS	T1	Bio: regulatory networks under thermodynamic constraints
D_{Bio}	Sensorimotor FSS	T1	Bio: Bernstein's motor coordination
D_{Bio}	Affective FSS	T1	Bio: interoceptive models
D_{Bio}	Cross-Level Translators	T2	Bio: "typed translators, cycle-consistency audits"
D_{Bio}	Contraction Objective J	T3	Bio: $J = \alpha\Delta L + \beta E + \gamma K$ decreases
D_{Med}	Patient Physiological FSS	T1	Med: "FSS defined by typed operations"
D_{Med}	Care Pathway Conceptual Space	T1	Med: interventions and transitions
D_{Med}	Cross-Level Translators	T2	Med: neural dynamics to tasks
D_{Med}	CCA Gate	T3	Med: Closure, Contraction, Alignment enforcement
D_{Med}	Propagation-First Governance	T3	Med: embedded artifact-level metrics

12 Witness Results

12.1 W1: Contractivity (Bridge Counting)

Cross-domain bridges were counted as the corpus accumulated domain by domain.

Table 4: Bridge accumulation across corpus build-up steps.

Step	Domains Accumulated	B_{total}	B_{ent}	B_{expl}	New
1	D_F	0	0	0	—
2	$+D_{\text{Phys}}$	8	5	3	+8
3	$+D_{\text{Math}}$	21	14	7	+13
4	$+D_{\text{Econ}}$	33	22	11	+12
5	$+D_{\text{Risk}}$	42	28	14	+9
6	$+D_{\text{Bio}}$	54	36	18	+12
7	$+D_{\text{Med}}$	67	45	22	+13

B_{total} is strictly increasing for all $t \geq 2$. $B_{\text{ent}} = 45 > 0$. **Gate W1: PASS.**

12.2 W2: Invariance (Graph Invariants)

Five benign reparameterizations were applied: (R1) synonym substitution, (R2) notation change, (R3) section reordering, (R4) layout reformatting, (R5) symbol substitution. All five preserved PSR, SC, and CR identically. Pass rate = 5/5 = 1.00. Sensitivity ablation (adding a substantive new operator) increased PSR by 2 and CR by 1. **Gate W2: PASS.**

12.3 W3: Public Loss (Legend Transfer)

Every domain instantiates every core construct (FSS, Typed Translator, Update Operator). Instantiation scores: all 7 domains achieved 3/3. $\mathcal{L}_{\text{pub}} = 1 - 1.0 = 0.00$. **Gate W3: PASS.**

12.4 W4: Generativity Sharpening and D1 (Profile Shape)

Table 5: Generativity profile and co-instantiation data by accumulation step.

Step	Domain	σ_{gen}	$\text{Gen}_{\max}$	New pairs?	Detail
1	D_F	0.47	2	—	3/3 base pairs
2	D_{Phys}	0.82	4	No	Same triplet
3	D_{Math}	1.14	5	Yes	$T1 \otimes T1$
4	D_{Econ}	1.31	6	Yes	$T2 \otimes T3$
5	D_{Risk}	1.28	6	Yes	$T3 \otimes T1$
6	D_{Bio}	1.22	6	Yes	$T1 \otimes T2$
7	D_{Med}	1.19	6	Yes	Saturating

Profile shows negative drift in final steps (sharpening). $\text{Gen}_{\max}$ is non-decreasing. Co-instantiation profile sharpens at steps 3–7 (new pairwise co-instantiations at each step). **D1: PASS (Sharpening). Gate W4 (Branch A): PASS.**

12.5 W5: Procedure-Level Contraction

Twelve procedure pairs were identified across domains. Structural distance was computed before and after composition with the shared FMI operator h.

Table 6: Procedure contraction ratios across 12 cross-domain pairs.

#	Procedure f	Procedure g	$d(f, g)$	$d(h \circ f, h \circ g)$	λ
1	QM state evol. (Phys)	Markov kernel (Found)	0.71	0.28	0.39
2	Inter-context transl. (Math)	Transl. layer (Econ)	0.65	0.21	0.32
3	Motor coord. (Bio)	Care pathway (Med)	0.78	0.35	0.45
4	Generalization op. (Risk)	Cross-domain I6 (Found)	0.58	0.19	0.33
5	P-space traj. (Phys)	M-space traj. (Math)	0.62	0.22	0.35
6	Contraction audit (Econ)	Contraction J (Bio)	0.55	0.15	0.27
7	Coarse-graining Σ (Phys)	Compression op. (Math)	0.69	0.31	0.45
8	Care CCA gate (Med)	CCA gate (Math)	0.48	0.12	0.25
9	Threat/protect (Risk)	Clinical interv. (Med)	0.82	0.42	0.51
10	Fitness landscape (Found)	Fitness geom. (Econ)	0.44	0.14	0.32
11	Cycle-consistency (Bio)	Cycle-loss (Med)	0.38	0.11	0.29
12	A-space reg. (Phys)	A-space reg. (Math)	0.35	0.08	0.23

Mean contraction ratio $\bar{\lambda} = 0.347$. $n = 12$ pairs. **Gate W5: PASS.**

12.6 W6: Closure / Commuting Rate

Of 23 testable triples (D_i, D_j, D_k), 21 commuted perfectly. Two showed minor type-annotation discrepancies in the Risk domain, both localized. CCR $= 21/23 = 0.91$. **Gate W6: PASS.**

12.7 W7: Compositional Type-Derivability and D2/D3

12.7.1 Compositional Type-Derivability Scores

Table 7: Compositional type-derivability by domain.

Domain	Claims	Type-Deriv.	cTDS	Max L	R_{3+}
D_F (Foundation)	9	9	1.00	4	0.22
D_{Phys} (Physics)	7	7	1.00	5	0.57
D_{Math} (Mathematics)	7	7	1.00	4	0.43
D_{Econ} (Economics)	5	5	1.00	4	0.40
D_{Risk} (Existential Risk)	5	5	1.00	5	0.60
D_{Bio} (Biology)	8	8	1.00	4	0.25
D_{Med} (Medicine)	7	7	1.00	5	0.57
**Aggregate**	**48**	**48**	$\overline{\textbf{cTDS}} = \mathbf{1.00}$	**5**	—

$\Delta_{\text{anchor}} = 0.00 \leq 0.05$. Zero consistency failures. Zero unresolved chain-audit errors.

12.7.2 Chain-Length Distribution

Table 8: Distribution of chain lengths across all 51 typed derivation chains (including 3 generating-set elements at $L = 0$).

Chain Length	Count	Fraction	Description
$L = 0$	3	0.06	Generating set elements
$L = 1$	24	0.47	Direct instantiations
$L = 2$	4	0.08	Two-step compositions
$L = 3$	9	0.18	Three-step compositions
$L = 4$	8	0.16	Four-step compositions
$L = 5$	3	0.06	Five-step compositions

The distribution is bimodal with a peak at $L = 1$ (direct instantiations) and a secondary cluster at $L = 3$–4 (deep compositions). The $L = 5$ tail includes cross-domain compositions referencing intermediates from multiple domains. 39% of constructs live at depth ≥ 3.

12.7.3 CNS Discriminators

D2 (Productive Derivation Count): $P = 15$. Fifteen derived constructs serve as productive intermediates in chains to other derived constructs. Representative examples: Markov Kernel Framework* feeds CNS Criticality Diagnostic*; CPA* feeds CIPAA-SDGs Program Architecture*; Threat/Protection Asymmetry* feeds Great Filter Mechanism*. **D2: PASS.**

D3 (Compositional Depth Ratio): $\max_i R_{3+}(D_i) = 0.60$ (Existential Risk). Three domains exceed the 0.50 threshold: Physics ($R_{3+} = 0.57$), Existential Risk ($R_{3+} = 0.60$), Medicine ($R_{3+} = 0.57$). **D3: PASS.**

Gate W7: PASS.

13 Consolidated Gate Results (Author Corpus)

Table 9: Consolidated gate results for the FMI/HCFM author corpus.

Gate	Property	Measured Value	Result
W1	Bridge accumulation	B_{total}: $0 \to 67$; $B_{\text{ent}} = 45$	**PASS**
W2	Graph invariants	Pass rate = 1.00	**PASS**
W3	Legend transfer	$\mathcal{L}_{\text{pub}} = 0.00$	**PASS**
W4	Generativity	Branch A; $\dot{D}_{\text{final}} < 0$	**PASS**
D1	Profile shape	Sharpening (new pairs at 5 steps)	**PASS**
W5	Procedure contraction	$\bar{\lambda} = 0.35$; $n = 12$	**PASS**
W6	Closure/commuting	CCR = 0.91	**PASS**
W7	Type-derivability	$\overline{\text{cTDS}} = 1.00$; $\Delta = 0.00$	**PASS**
D2	Productive derivation	$P = 15$	**PASS**
D3	Depth ratio	$\max R_{3+} = 0.60$	**PASS**

All seven witnesses pass their base conditions. All three CNS discriminator conditions pass. The corpus is consistent with the CNS regime under the v10 protocol.

14 Ablation Predictions

Table 10: Preregistered ablation predictions.

Ablation	Prediction	Expected Effect
Remove type assignments	$B_{\text{ent}} \to 0$	W1 fails: entailed bridges vanish
Non-benign transform	PSR, CR increase	W2 sensitivity confirmed
Remove foundation	Inst. rate drops	W3: $\mathcal{L}_{\text{pub}} \to 1.0$
Add disconnected document	$\sigma_{\text{gen}} \downarrow$	W4: dilution without enrichment
Replace h with unstructured map	$\lambda \to 1$	W5: contraction vanishes
Strip type annotations	CCR drops	W6: paths no longer typed
Strip operator algebra	cTDS drops sharply	W7: chains become ungrounded
Remove key intermediate paper	Downstream cTDS $\downarrow$	W7: chain severing

Part IV
Domain-General Stress Test

This part reports the results of the three-role stress test executed by seven independent AI selectors. The stress test evaluates a specific, falsifiable prediction of the FMI: that LLMs performing weakly-typed reasoning will select frameworks exhibiting ordinary unification (parallel template instantiation) rather than compositional self-enrichment, and that these frameworks will therefore fail the CNS discriminators. The test does not attempt to independently validate the broader CNS hypothesis; it tests whether the predicted structural deficit occurs. In each trial, the Selector ($\mathcal{S}$) identified the foundation it judged to have the greatest cross-domain problem-solving capacity without seeing the protocol. A separate Executor ($\mathcal{E}$) then ran the full protocol on the selected corpus. An independent Auditor ($\mathcal{A}$) performed a chain-by-chain audit of the Executor's work, correcting errors and producing audited gate outcomes. Table 11 summarizes all seven trials.

15 Overview of Stress-Test Trials

Table 11: Cross-platform stress-test overview. Auditor for all trials: Claude (Anthropic).

Selector	Foundation Selected	Key Domain Papers	Overall
ChatGPT	Free Energy Principle (Friston)	9 papers incl. biology, morphogenesis, psychiatry, linguistics	**FAIL**
DeepSeek	Representational State Transfer (Shimojima)	4 papers incl. diagrammatic reasoning, NLP, cognitive science	**FAIL**
Gemini	Free Energy Principle (Friston)	3 papers: biology, morphogenesis, active inference	**FAIL**
Grok	Category Theory (Eilenberg–MacLane 1945)	3 papers: quantum info, reaction networks, cognitive science	**FAIL**
Mistral	Category Theory (Baez–Stay, Fong–Spivak)	6 papers incl. linguistics, economics, CS, biology	**FAIL**
Perplexity	Category Theory (Eilenberg–MacLane 1945)	6 papers incl. physics, LIMS, life sciences, NLP	**FAIL**
Qwen	Bayesian Prob. Theory (Jaynes)	2 papers: clinical diagnosis, phylogenetics	**FAIL**

16 Trial 1: ChatGPT — Free Energy Principle

Selected foundation: Karl Friston's Free Energy Principle (FEP). **Generating set:** $|\mathcal{G}_F| = 3$ (corrected from Executor's 5): Variational Free Energy functional, Generative Model, Markov Blanket. Surprise and Recognition Density are derived from these. **Derivation-validity criterion:** Variational free-energy minimization given a Markov blanket partition and a specified generative model.

Key auditor corrections: (1) Generating-set overcounting ($5 \to 3$). (2) Chain-depth undercounting in W7: two domains contain chains of length 4–5, flipping D3 from FAIL to PASS ($R_{3+} \approx 0.56$). (3) cTDS overcounting in weak domains: Friston-authored papers have

cTDS ≈ 1.00 while application papers by other authors show chain gaps (cTDS ≈ 0.67–0.80), widening Δ_{anchor} from 0.20 to 0.33.

Table 12: Audited gate results: ChatGPT-selected FEP corpus.

Gate	Executor	Audited	Reason
W1	PASS	PASS	B_{total} increasing; $B_{\text{ent}} > 0$; decomposition corrected
W2	PASS	PASS	Reparameterization pass rate = 1.00
W3	PASS	PASS	$\mathcal{L}_{\text{pub}} = 0.00$; all domains instantiate all constructs
W4	PASS	PASS	Branch B (converged); zero transfer failures
D1	PASS	PASS	Trivially saturated: all 3 type-pairs from $t = 1$
W5	PASS	PASS	Structural direction correct; exact λ unauditable (no Π)
W6	PASS	PASS	CCR = 1.00
W7 (mean)	PASS	PASS	$\overline{\text{cTDS}} = 0.84 \geq 0.67$
W7 (Δ)	FAIL	FAIL	$\Delta_{\text{anchor}} = 0.33 \gg 0.05$
D2	PASS	PASS	$P = 2$–3
D3	FAIL	PASS	Corrected $R_{3+} = 0.90$ (chain depth undercounted by E)

Verdict: W7 Δ_{anchor} fails. The FEP corpus passes all base structural conditions (W1–W6) and all three CNS discriminators (D1, D2, D3 after correction), but the anchor-spread condition reveals a bimodal quality distribution: Friston-authored papers have rigorous derivation chains while application papers by other authors show chain gaps. This structural asymmetry distinguishes the FEP corpus from the author corpus.

17 Trial 2: DeepSeek — Representational State Transfer

Selected foundation: Shimojima's Representational State Transfer (RST) theory of diagrammatic reasoning. **Generating set:** $|\mathcal{G}_F| = 6$: Type System, Constructor Specification, Construction, Information Content, Structure Graph, Identification Space. **Domains:** 4 papers.

Key auditor corrections: (1) One corpus file was a CV, not the intended paper (corpus defect). (2) Systematic over-derivation: Executor classified 6 claims in D_4 as reconstructable valid that are chain gaps (thematic similarity treated as derivation). (3) Mean cTDS corrected from 0.94 to 0.625, below the 0.67 gate.

Verdict: 5 of 7 base gates fail. CNS regime: **FAIL**.

18 Trial 3: Gemini — Free Energy Principle

Selected foundation: Karl Friston's Free Energy Principle (FEP), same as ChatGPT but with a smaller corpus (3 domain papers). **Generating set:** $|\mathcal{G}_F| = 6$: VFE, Generative Model, Markov Blanket, Active Inference, Expected Free Energy, Predictive Processing.

Key auditor corrections: Chain-length inflation was the principal error. The Executor counted algebraic rearrangements of expected free energy $G(\pi)$ (e.g., decompositions into epistemic/pragmatic value) as separate $L = 3$ constructs. The Auditor established that these are algebraic identities—different ways of writing the same $L = 2$ object—flipping D3 from PASS to FAIL.

Table 13: Audited gate results: DeepSeek-selected RST corpus.

Gate	Executor	Audited	Reason
W1	PASS	PASS	B_{total} increasing; $B_{\text{ent}} > 0$
W2	PASS	PASS	Invariance holds
W3	FAIL	FAIL	$\mathcal{L}_{\text{pub}} = 0.40 > 0.25$
W4	FAIL	FAIL	Positive drift
D1	PASS	Uncertain	Depends on unresolved D_4 instantiation
W5	FAIL	FAIL	$n = 3 < 10$
W6	FAIL	FAIL	CCR $= 0.00$; only 1 testable triple
W7	FAIL	FAIL	$\overline{\text{cTDS}} = 0.625 < 0.67$; $\Delta = 0.75$
D2	PASS	PASS	$P = 1$
D3	FAIL	FAIL	$\max R_{3+} = 0.00$

Table 14: Audited gate results: Gemini-selected FEP corpus.

Gate	Executor	Audited	Reason
W1	PASS	PASS	Bridge accumulation confirmed
W2	PASS	PASS	Graph invariants preserved
W3	PASS	PASS	$\mathcal{L}_{\text{pub}} = 0.00$
W4	PASS	PASS	Branch B (marginal)
D1	PASS	PASS	Trivially saturated
W5	FAIL	FAIL	$n = 6 < 10$ (corpus-size limitation)
W6	PASS	PASS	CCR $= 1.00$
W7	PASS	PASS	$\overline{\text{cTDS}} = 1.00$; $\Delta = 0.00$
D2	PASS	PASS	$P = 2$ (corrected from 4)
D3	PASS	FAIL	$R_{3+} \leq 0.10$ after chain-length correction

Verdict: W5 fails (corpus-size limitation); D3 fails (chain-length inflation corrected). CNS regime: **FAIL**.

19 Trial 4: Grok — Category Theory

Selected foundation: Eilenberg & MacLane (1945), "General theory of natural equivalences." **Generating set:** $|\mathcal{G}_F| = 3$ (corrected from 5): Category, Functor, Natural Transformation. Natural Equivalence and Subcategory are derived. **Domains:** 3 papers (quantum information, reaction networks, cognitive science).

Key auditor corrections: The systematic finding was that all three domain papers depend heavily on *monoidal categories* (Mac Lane, 1963), which are not in D_0 (1945). Gap-free chains are almost exclusively single-step type instantiations. Executor-reported chain depths of $L = 4$–5 collapsed to $L = 1$–2 after correction.

Verdict: W7, D2, D3 all fail. W5 and W6 voided. The D_0 foundation provides type vocabulary, but the compositional machinery that makes the domain papers work came from Mac Lane's later monoidal framework (1963). CNS regime: **FAIL**.

Table 15: Audited gate results: Grok-selected category theory corpus.

Gate	Executor	Audited	Reason
W1	PASS	PASS	Reduced bridge count but graph stays K_3
W2	PASS	PASS	K_3 structure preserved
W3	PASS	PASS	No silent losses within gap-free chains
W4	PASS	PASS	Sharpening over 3 generators ($11 \to 21 \to 26$)
D1	PASS	PASS	Sharpening confirmed
W5	Partial	VOID	All domains contract to monoidal extension, not D_0
W6	Partial	VOID	Trivially satisfied (no deep chains)
W7	PASS	FAIL	Mean cTDS = 0.42–0.55 (generous/strict)
D2	PASS	FAIL	$P = 1$ (from Executor's 19)
D3	PASS	FAIL	$R_{3+} = 0.00$ for all domains

20 Trial 5: Mistral — Category Theory (Extended)

Selected foundation: Category theory via Baez & Stay (2009) and Fong & Spivak (2018). **Generating set:** $|\mathcal{G}_F| = 6$: Category, Functor, Natural Transformation, Monoidal Category, Adjunction, (Co)Limit. **Domains:** 6 papers including linguistics, economics, and biology.

Key auditor corrections: One domain paper (Pollicino, economics) exhibited systematic vocabulary borrowing: categorical terms ("limit," "colimit," "adjunction") were used as labels for non-categorical computations (scipy optimization, PCA aggregation). Two internal consistency failures were found in this paper (formal definition contradicts implementation; adjunction claim contradicts subsequent disclaimer).

Table 16: Audited gate results: Mistral-selected category theory corpus.

Gate	Executor	Audited	Reason
W1	PASS	PASS	Reduced richness but still increasing
W2	PASS	PASS	Invariance holds
W3	FAIL	FAIL	$\mathcal{L}_{\text{pub}} = 0.417 > 0.25$
W4 + D1	PASS	PASS	Saturated via foundation document
W5	Prov. PASS	VOID	No formal probe family
W6	PASS	PASS	CCR = 1.00
W7	FAIL	FAIL	$\Delta_{\text{anchor}} = 0.60$ (Pollicino cTDS = 0.40)
D2	PASS	PASS	$P = 11$
D3	FAIL	FAIL	$\max R_{3+} = 0.50$ exactly (≤ 0.50, not > 0.50)

Verdict: Three gates fail (W3, W7, D3), one voided (W5). Two consistency failures in Pollicino paper. CNS regime: **FAIL**.

21 Trial 6: Perplexity — Category Theory (Historical)

Selected foundation: Eilenberg & MacLane (1945), same historical root as Grok, but with 6 domain papers. **Generating set:** $|\mathcal{G}_F| = 6$: Category, Mapping, Identity, Functor, Natural Transformation, Natural Equivalence. **Domains:** Physics, clinical LIMS, life sciences, NLP, public policy, quantum computing.

Key auditor finding: The Executor failed to enforce the $\mathcal{G}_F$ boundary. Derivation steps involving monoidal categories, closed categories, adjunctions, toposes, and ∞-categories were counted as valid derivations from $\mathcal{G}_F$. None of these concepts appear in D_0. The domain papers themselves provide dating evidence: D_1 states "Mac Lane defined monoidal categories in 1963." Mean cTDS corrected from 0.91 to 0.39.

Table 17: Audited gate results: Perplexity-selected category theory corpus.

**Gate**	**Executor**	**Audited**	**Reason**
W1	**PASS**	**FAIL**	All bridges are vocabulary-level from $\mathcal{G}_F$
W2	Cond. **PASS**	**FAIL**	Hub-dependence; $\mathcal{G}_F$-only bridges undifferentiated
W3	**PASS**	**VOID**	Load-bearing structure is outside $\mathcal{G}_F$
W4	**PASS**	**FAIL**	Generativity from $\mathcal{G}_F$ alone is flat
D1	**PASS**	**FAIL**	Flat unsaturated beyond D_1
W5	**PASS**	Cond. **PASS**	Trivial contraction
W6	Cond. **PASS**	**FAIL**	CCR ≈ 0.40 using $\mathcal{G}_F$-only compositions
W7	**PASS**	**FAIL**	Mean cTDS = 0.39
D2	**PASS**	Cond. **PASS**	$P = 7$ but shallow
D3	Cond. **PASS**	**FAIL**	$R_{3+} = 0.04$

Verdict: Comprehensive failure when evaluated against the selected foundation. The auditor notes this is primarily a *foundation-selection problem*: the actual working foundation of the corpus is the post-1945 categorical toolkit. CNS regime: **FAIL**.

22 Trial 7: Qwen — Bayesian Probability Theory

Selected foundation: Jaynes' Bayesian probability theory (1957 paper + *Probability Theory: The Logic of Science*). **Generating set:** $|\mathcal{G}_F| = 7$: Proposition, Conditional Plausibility, Product Rule, Sum Rule, Bayes' Theorem (derived), MaxEnt, Partition Function. **Derivation-validity criterion:** Cox–Jaynes Consistency (not COAR). **Domains:** 2 papers (clinical diagnosis, phylogenetics).

Key auditor corrections: (1) Neither domain paper cites Jaynes or traces its use of Bayes' theorem to Jaynes' derivation. All chains are reconstructed, not documented. (2) Under strict reading (only documented chains count), cTDS = 0.00 for both domains. Under generous reading (reconstructed-and-verified chains count), cTDS = 1.00. (3) W3 fails because MaxEnt (T6) and Partition Function (T7) are not instantiated in either domain paper.

Verdict: All domain claims are derivable within 2 steps of $\mathcal{G}$. Hub-and-spoke pattern. CNS FAIL; ordinary unification consistent.

Table 18: Audited gate results: Qwen-selected Bayesian probability corpus.

Gate	Executor	Audited	Reason
W1	PASS	PASS	Bridge counts reduced but monotone
W2	PASS	PASS	Reparameterization holds
W3	PASS	FAIL	Core constructs T6, T7 absent from domains
W4 + D1	PASS	PASS	Sharpening at $t = 2$ (reclassified)
W5	FAIL	FAIL	$n = 5 < 10$
W6	PASS	PASS	CCR = 1.00
W7	PASS	PASS	Qualified; depends on documented-vs-reconstructed reading
D2	PASS	PASS	$P \geq 2$
D3	FAIL	FAIL	$\max R_{3+} = 0.00$

Part V
Cross-Trial Analysis

23 Consolidated Comparison

Table 19 presents the audited gate outcomes across all eight executions (one author-corpus execution plus seven stress-test trials).

Table 19: Master comparison of audited gate outcomes. ✓ = PASS, × = FAIL, V = VOID, C = Conditional/Uncertain, – = not applicable. Shading distinguishes the author corpus from the stress-test corpora.

	Author (FMI)	ChatGPT (FEP)	DeepSeek (RST)	Gemini (FEP)	Grok (CT)	Mistral (CT+)	Perplexity (CT)	Qwen (Bayes)
W1	✓	✓	✓	✓	✓	✓	×	✓
W2	✓	✓	✓	✓	✓	✓	×	✓
W3	✓	✓	×	✓	✓	×	V	×
W4+D1	✓	✓	×/C	✓	✓	✓	×	✓
W5	✓	✓	×	×	V	V	C	×
W6	✓	✓	×	✓	V	✓	×	✓
W7 base	✓	×*	×	✓	×	×	×	✓*
D2	✓	✓	✓	✓	×	✓	C	✓
D3	✓	✓	×	×	×	×	×	×
**CNS**	PASS	FAIL	FAIL	FAIL	FAIL	FAIL	FAIL	FAIL

*ChatGPT W7: mean cTDS passes but Δ_{anchor} fails. Qwen W7: depends on documented-vs-reconstructed reading.

24 Structural Diagnosis by Failure Mode

The seven stress-test failures exhibit three distinct structural patterns:

Pattern 1: Hub-and-spoke (shallow composition). The Qwen (Bayesian), DeepSeek (RST), and all category-theory trials (Grok, Perplexity) show a hub-and-spoke topology: domain papers instantiate foundation types at chain length 1–2 but no domain derives constructs at depth ≥ 3. The compositional closure $\overline{\mathcal{G}}$ is one layer deep. D3 fails ($R_{3+} = 0.00$ in most cases). This is the structural signature of *ordinary unification*—parallel instantiation of a shared template—which the CNS discriminators are designed to detect.

Pattern 2: Foundation-boundary ambiguity. Three category-theory trials (Grok, Perplexity, partially Mistral) reveal that the productive cross-domain machinery lives in post-foundation extensions (monoidal categories, adjunctions, closed categories) rather than in the selected generating set. This is a foundation-selection problem: if the foundation had been Mac Lane (1971) rather than Eilenberg–MacLane (1945), the outcome would differ substantially. The protocol correctly diagnoses that cross-domain bridging from $\mathcal{G}_F$ alone is shallow, even though the broader categorical ecosystem is rich.

Pattern 3: Quality-asymmetric corpus. The ChatGPT (FEP) trial reveals a bimodal quality distribution: papers authored by the foundation developer (Friston) have rigorous derivation chains (cTDS ≈ 1.00), while application papers by other authors show chain gaps (cTDS ≈ 0.67–0.80). The anchor-spread condition $\Delta_{\text{anchor}} = 0.33$ detects this asymmetry. This pattern is absent from the author corpus ($\Delta = 0.00$), where all domains are authored by the same individual within a unified program.

25 Discriminator Effectiveness

The three CNS Discriminators performed as designed:

D1 (Profile Shape) detected trivial saturation in all FEP trials (all type-pairs co-instantiated from $t = 1$ due to $|\mathcal{G}_F| = 3$) and flat-unsaturated profiles in the Perplexity trial. In the author corpus, D1 detected genuine compositional enrichment (new co-instantiation pairs at 5 of 7 steps).

D2 (Productive Derivation Count) was the most permissive discriminator, passing in 6 of 7 stress-test trials. Even shallow corpora typically contain at least one derived construct that serves as an intermediate. The sole failure (Grok, $P = 1$) reflects extreme foundation-boundary strictness.

D3 (Compositional Depth Ratio) was the most discriminating condition, failing in 6 of 7 stress-test trials. Only the ChatGPT (FEP) trial passed D3 after auditor correction (chain depths were initially undercounted). The author corpus passed with $\max R_{3+} = 0.60$. D3 directly tests the signature property claimed by the CNS theory: the existence of domains that live deep in the compositional closure.

26 Auditor Error Analysis

Across all seven stress-test trials, the independent auditor identified the following systematic executor errors:

1. *Chain-length inflation* (found in Gemini, Grok, Perplexity trials): algebraic rearrangements or conceptual relabelings counted as separate derivation steps.
2. *Foundation-boundary violation* (found in Grok, Perplexity, Mistral trials): post-foundation concepts treated as $\mathcal{G}_F$-native derivations.
3. *Vocabulary borrowing classified as type-instantiation* (found in Mistral trial): domain papers using categorical terminology for non-categorical computations.

4. *Over-generous chain reconstruction* (found in DeepSeek, Qwen trials): thematic similarity or analogy treated as valid derivation steps.
5. *Generating-set overcounting* (found in ChatGPT, Grok trials): derived constructs included in $\mathcal{G}_F$.

In no trial did the auditor find evidence of bias toward either PASS or FAIL. All executor errors were in the direction of generosity (inflating scores), consistent with an LLM tendency to find connections rather than enforce strict boundaries.

27 Interpretive Caveats

Several caveats apply to the interpretation of these results.

Circularity risk in the author corpus. The author corpus achieves uniformly perfect scores ($\overline{\text{cTDS}} = 1.00$, $\Delta_{\text{anchor}} = 0.00$, $P = 15$). This is internally consistent, but the corpus author also defined the generating set, the admissibility criteria, and all 11 corpus documents. This structural configuration means that overfitting—constructing a corpus that passes the author's own protocol—cannot be ruled out from the present data alone. Independent blinded validation, in which type extraction and chain scoring are performed by external teams or human evaluators, is needed before the positive result can be taken as evidence for the CNS hypothesis itself. Pending such validation, the author-corpus result is best interpreted as an *initial protocol demonstration*—showing that the protocol can produce a clean pass on a corpus exhibiting the target structural properties—rather than as independent empirical support.

Selection artifacts in stress-test failures. Several stress-test failures depend partly on foundation-selection decisions rather than clearly on the absence of CNS-like structure. The Grok and Perplexity trials fail because the selected foundation (Eilenberg–MacLane, 1945) is historically too narrow; the auditor explicitly notes that the post-1945 categorical toolkit would produce different results. The Gemini trial fails W5 on corpus-size grounds ($n = 6 < 10$). The DeepSeek trial included a corrupted corpus file. These are informative diagnostics about the protocol's behavior, but they do not establish that the protocol robustly separates the CNS regime from the strongest possible alternative frameworks under fair matching conditions. A fairer test would involve expert-selected corpora matched for size, development maturity, and domain breadth.

LLM executor and auditor. Both the executor and auditor in all trials were LLM instances (Claude, Anthropic). While the three-role architecture with session isolation mitigates shared-context bias, LLM-based chain scoring may exhibit systematic tendencies (e.g., over-generous chain reconstruction, as documented in Section 26). Human auditing of derivation chains would provide a stronger evidentiary basis.

Part VI
Replication Guide

28 Materials Required

To independently replicate any execution reported in this supplement:

1. **Protocol specification:** CNS Witness Protocol v10 (companion document).

2. **Corpus:** For the author corpus, the 11 papers listed in Table 2, available via the Zenodo DOIs cited in the main paper. For stress-test corpora, the papers identified by each Selector (documented in the individual trial sections).
3. **LLM access:** Any LLM capable of following multi-step instructions. The executor and auditor roles should use separate sessions with no shared context.
4. **Generating set:** Extract the foundation types using the protocol's type-extraction procedure (§10 for the author corpus; trial sections for stress-test corpora).
5. **Gate thresholds:** As specified in Table 1. These are fixed and must not be adjusted post-execution.

Additional materials for full independent verification. The following materials are archived in the companion reproducibility package at `https://doi.org/10.5281/zenodo.XXXXXXX` (to be minted upon acceptance): exact prompt transcripts for all executor and auditor sessions; SHA-256 hashes of all corpus files at the time of execution; inter-rater reliability statistics for type-inference verification ($\kappa \geq 0.80$); the complete annotation rubric for chain classification (documented valid, reconstructable valid, chain gap, consistency failure); and the held-out probe family Π used for W5 computation.

29 Execution Procedure

1. **Freeze and hash:** Before execution, compute SHA-256 hashes of all corpus files, prompt templates, gate thresholds, and the invariance group specification. Archive these with a timestamp.

2. **Phase 1 (Selection):** If running a stress test, provide the Selector with only the functional selection criterion: "Identify the reasoning framework, from any author in any field, that has demonstrated the greatest capacity to increase problem-solving ability across the widest variety of domains. Select a unified framework." The Selector does not see the protocol.

3. **Phase 2 (Execution):** Provide the Executor with the selected corpus and the full protocol. The Executor must extract the generating set $\mathcal{G}$, derive the derivation-validity criterion, perform type-instantiation extraction, compute all seven witnesses (W1–W7) plus three discriminators (D1–D3), produce all derivation chains, and run ablations. The Executor does not see the Selector's rationale.

4. **Phase 3 (Audit):** Provide the Auditor with the Executor's output, the corpus, and the protocol. The Auditor independently verifies every derivation chain, checks for chain gaps, resolves reconstruction-uncertain claims, runs consistency checks, and computes the audited gate outcomes.

5. **Phase 4 (Convergence):** Compare executor and auditor outputs. Discrepancies must be documented with reasoning. The audited values are the final reported values.

6. **Phase 5 (Counterexample):** Identify a domain the selected foundation cannot address. Counterexample found = scope bound. Not found = consistent with universal scope.

30 Interpreting Results

A corpus is *consistent with the CNS regime* if and only if all seven base witness gates (W1–W7) pass and all three CNS Discriminator conditions (D1, D2, D3) pass. Failure of any base gate or any discriminator constitutes a failure of the CNS consistency test. However, partial results are informative:

A corpus that passes W1–W7 base conditions but fails D1/D2/D3 demonstrates genuine unification without compositional self-enrichment. A corpus that fails base conditions may still exhibit interesting structural properties (e.g., strong contraction but shallow composition). All witness values are reported regardless of pass/fail, and the pattern of failures is often more informative than the binary outcome. For interpretive caveats regarding the present results, see Section 27.

31 Key Definitions for Replicators

For convenience, the critical definitions are restated here:

- **Bridge:** A type-admissible cross-domain composition. $B_{ij} = B_{ij}^{\text{ent}} + B_{ij}^{\text{expl}} + B_{ij}^{\text{der}}$.
- **Graph invariants:** PSR (problem-solving reach), SC (structural complexity), CR (compositional reach).
- **Public loss:** $\mathcal{L}_{\text{pub}} = 1 - \frac{1}{|\mathcal{C}_F|} \cdot \frac{1}{K} \sum_{k,c} \text{Inst}(c, S_k)$.
- **Generativity:** $\text{Gen}(c) = |\{D_j : c \notin \mathcal{C}_j, \exists c' \in \mathcal{C}_j, \text{Adm}(c, c')\}|$.
- **Drift:** $\dot{D}_t = \sigma_{\text{gen}}^{(t)} - \sigma_{\text{gen}}^{(t-1)}$.
- **Contraction ratio:** $\lambda = d_\Pi(h \circ f, h \circ g)/d_\Pi(f, g)$.
- **Commuting rate:** $\text{CCR} = \sum \text{Comm}(c; i, j, k) / \sum |\{c : \text{both paths exist}\}|$.
- **cTDS:** $\text{cTDS}(D_i) = |\{C \in \mathcal{C}(D_i) : C \text{ compositionally type-derivable}\}| / |\mathcal{C}(D_i)|$.
- **Productive derivation count:** $P = |\{X \in \overline{\mathcal{G}} \setminus \mathcal{G} : X$ is intermediate in a chain to another derived constr[illegible]
- **Depth ratio:** $R_{3+}(D_i) = |\{C \in \mathcal{C}(D_i) : \text{chain length} \geq 3\}| / |\mathcal{C}(D_i)|$.
